# Gestational and early postnatal hypothyroidism alters VGluT1 and VGAT bouton distribution in the neocortex and hippocampus, and behavior in rats

**DOI:** 10.3389/fnana.2015.00009

**Published:** 2015-02-17

**Authors:** Daniela Navarro, Mayvi Alvarado, Francisco Navarrete, Manuel Giner, Maria Jesus Obregon, Jorge Manzanares, Pere Berbel

**Affiliations:** ^1^Departamento de Histología y Anatomía, Facultad de Medicina, Universidad Miguel HernándezAlicante, Spain; ^2^Instituto de Neuroetología, Universidad VeracruzanaXalapa, Veracruz, México; ^3^Instituto de Neurociencias de Alicante, Universidad Miguel Hernández and Consejo Superior de Investigaciones CientíficasAlicante, Spain; ^4^Instituto de investigaciones Biomédicas, Consejo Superior de Investigaciones Científicas and Universidad Autónoma de MadridMadrid, Spain

**Keywords:** cerebral cortex, iodine deficiency, attention deficit-hyperactivity disorder, autism, schizophrenia, prepulse inhibition, anxiety-like, seizures

## Abstract

Thyroid hormones are fundamental for the expression of genes involved in the development of the CNS and their deficiency is associated with a wide spectrum of neurological diseases including mental retardation, attention deficit-hyperactivity disorder and autism spectrum disorders. We examined in rat whether developmental and early postnatal hypothyroidism affects the distribution of vesicular glutamate transporter-1 (VGluT1; glutamatergic) and vesicular inhibitory amino acid transporter (VGAT; GABAergic) immunoreactive (ir) boutons in the hippocampus and somatosensory cortex, and the behavior of the pups. Hypothyroidism was induced by adding 0.02% methimazole (MMI) and 1% KClO_4_ to the drinking water starting at embryonic day 10 (E10; developmental hypothyroidism) and E21 (early postnatal hypothyroidism) until day of sacrifice at postnatal day 50. Behavior was studied using the acoustic prepulse inhibition (somatosensory attention) and the elevated plus-maze (anxiety-like assessment) tests. The distribution, density and size of VGluT1-ir and VGAT-ir boutons in the hippocampus and somatosensory cortex was abnormal in MMI pups and these changes correlate with behavioral changes, as prepulse inhibition of the startle response amplitude was reduced, and the percentage of time spent in open arms increased. In conclusion, both developmental and early postnatal hypothyroidism significantly decreases the ratio of GABAergic to glutamatergic boutons in dentate gyrus leading to an abnormal flow of information to the hippocampus and infragranular layers of the somatosensory cortex, and alter behavior in rats. Our data show cytoarchitectonic alterations in the basic excitatory hippocampal loop, and in local inhibitory circuits of the somatosensory cortex and hippocampus that might contribute to the delayed neurocognitive outcome observed in thyroid hormone deficient children born in iodine deficient areas, or suffering from congenital hypothyroidism.

## Introduction

Severe to mild thyroid hormone deficiency during gestation and early postnatal ages (less than 3 years old) causes a wide spectrum of disorders, ranging from stillbirths, miscarriages, congenital anomalies, deafness, neurocognitive delay, and mental retardation (Porterfield and Hendrich, 1993; Hetzel, 1994; Chan and Kilby, 2000; Ahmed et al., 2008; Morreale de Escobar et al., 2008; Berbel and Bernal, 2010; Berbel and Morreale de Escobar, 2011). In 1990, it was estimated that 1600 million people are exposed to iodine deficiency worldwide (about 25% of the world population of which 11 million suffered from overt cretinism (the most extreme form of mental retardation due to iodine deficiency) and 43 million people were affected by some degree of mental impairment (Glinoer and Delange, 2000). Epidemiological studies have shown low IQ and neurological alterations in children from mildly iodine deficient mothers suffering from hypothyroxinemia, which may affect 25–40% of pregnant women living in mild-moderately iodine deficient countries (Haddow et al., 1999; Pop et al., 2003; Vermiglio et al., 2004; Kasatkina et al., 2006; Kooistra et al., 2006; Berbel et al., 2009; Suárez-Rodríguez et al., 2012; Zimmermann, 2013).

Recently, up to 552 genes that play a key role in cortical maturation at the end of gestation have been found to be regulated by L-triiodothyronine (T3) at the transcriptional level (Morte et al., 2010; Chatonnet et al., 2015). Important for the development of cortical connections are genes that code for: Nefh, Nefl and Nefm (neurofilament proteins); Slit1, Slit2, Nos1, BNDF, Camk4, and Creb (involved in bifurcation and growth of neural processes); Sema3B, Slit1 and Slit2 (guiding axons); and VGluT1 (vesicular glutamate transporter 1) (Morte et al., 2010). The role of thyroid hormones is relevant in the regulation of Camk4-Creb and Erk1/2-Creb pathways (Berbel et al., 2010, 2014; Morte et al., 2010; Navarro et al., 2014) which control fundamental phases of corticogenesis (Berbel et al., 2007) and cerebral cortex function (Carlezon et al., 2005; Navarro et al., 2014). In the central nervous system, the Camk4-Creb pathway is active in neurons, since Camk4 is not expressed in glial cells (Watterson et al., 2001; Murray et al., 2009; Navarro et al., 2014). There is strong evidence that the Camk4-Creb pathway is involved in the expression of the *FMR1* gene which codes for the fragile X mental retardation protein (FMRP) (Wang et al., 2009, 2012; Waltes et al., 2014). The lack of FMRP causes the fragile X syndrome (FXS) which is the most common cause of inherited mental retardation and autism spectrum disorders (ASD; Krueger and Bear, 2011). In response to the metabotropic glutamate receptor (mGluR) activation, FMRP mediates the activity-dependent dendritic mRNA transport and translation (Bagni and Greenough, 2005; Kao et al., 2010; Tatavarty et al., 2012). Current evidence suggests that FMRP and the brain-derived neurotrophic factor (BDNF) may regulate each other and alterations in BDNF expression modify the phenotype of FXS and ASD (Nishimura et al., 2007; Castrén and Castrén, 2014).

The effect of thyroid hormones on the organization and function of the cerebral cortex has been studied since the pioneering studies of Eayrs and cols (Eayrs and Taylor, 1951; Eayrs, 1955; see also recent reviews by Zoeller and Rovet, 2004; Morreale de Escobar et al., 2008; Koromilas et al., 2010; Berbel and Morreale de Escobar, 2011; Berbel et al., 2014). Recent studies have confirmed Eayrs' results and reported new data such as alterations in (i) the size of thalamic terminal arbors in the somatosensory cortex, (ii) the density of parvalbumin immunolabeled terminals in the auditory cortex of developmental hypothyroid rats (Berbel et al., 1996; Ausó et al., 2004), and (iii) the organization of commissural and thalamo-cortical connections (Ausó et al., 2001; Berbel et al., 2007). Developmental and postnatal hypothyroidism alters the structure and function of the hippocampus (Rami et al., 1987; Lavado-Autric et al., 2003; Venero et al., 2005; Gilbert and Sui, 2006; Opazo et al., 2008; Alzoubi et al., 2009; Sawano et al., 2013; Berbel et al., 2014; Wang et al., 2014). In early postnatal hypothyroid rats, a decrease has been reported in the density of parvalbumin-positive neurons in the hippocampus (Gilbert et al., 2007). In fetuses deprived of maternal thyroid hormones late in pregnancy (LMH pups), a 43% reduction of the Zn-positive area (labeling mossy fibers boutons) in the CA3 stratum lucidum was observed (Berbel et al., 2010). These studies suggest an alteration in the excitatory to inhibitory ratio in the neocortex and hippocampal formation of hypothyroid rats. Furthermore, control (C) and LMH pups were tested at P39 for aversive memory retrieval using a one-trial, step-down inhibitory avoidance task in which step-down latencies (to a ceiling of 3 min) at 1 (for the assessment of short-term memory), 3 and 24 h (for the assessment of long-term memory) were measured after training. In the 1 h test, step-down latencies were 25% lower in LMH than in C pups, indicating short-term altered memory consolidation in LMH pups (Berbel et al., 2010). In humans, the most commonly affected encephalic areas associated with childhood and adolescent psychiatric disorders are the frontal and associative areas of the neocortex, the limbic system, the striatum and the cerebellar cortex (Goodman et al., 2014). The aberrant development of the limbic system, which includes the hippocampal formation, amygdala, mammillary body, anterior cingulate gyrus and septum (Bauman and Kemper, 2005; Amaral et al., 2008; Goodman et al., 2014) and neocortex is associated with the pathogenesis and phenotypic expression of childhood psychiatric disorders such as ASD (Bauman and Kemper, 2005; Román et al., 2013; Berbel et al., 2014), attention deficit-hyperactivity disorder (ADHD) (Li et al., 2014a,b), Alzheimer's disease (Llorens-Martín et al., 2014) and schizophrenia (Santos et al., 2012). Neurons in the limbic areas of autistic humans show reduced cell size and a higher cell packing density than controls (Bauman and Kemper, 2005).

It remains less well known, particularly during gestation and at earlier postnatal ages, how thyroid hormones might affect the balance of excitatory and inhibitory inputs of the neocortex and of hippocampal formation, which include the dentate gyrus (DG) and the Cornu Ammonis (CA) (see Materials and Methods; Amaral and Witter, 1995), and consequently the hippocampal intrinsic circuitry. These data might help to gain a better understanding of the neuropathology of neurological diseases, comorbid to ASD, ADHD and schizophrenia. Our aim has been to study the effect of gestational and early postnatal hypothyroidism on the distribution, ratio and size of vesicular glutamate transporter type 1 (VGluT1; labeling glutamatergic) and that of vesicular inhibitory amino acid transporter (VGAT; labeling GABAergic) immunoreactive (ir) boutons in the hippocampal formation and somatosensory cortex of rats. Changes in the balance of excitatory and inhibitory inputs in the neocortex and hippocampus may affect attention and exploratory behaviors in which the neocortex and hippocampus are involved. In particular, we have studied altered attention deficit, using the acoustic prepulse inhibition test, and anxiety-like behavior, using the elevated plus-maze test.

## Materials and methods

### Ethics statement

Care of the animals and drug administration were performed under veterinary control according to European Union Directive 86/609/EEC and with approval from the Ethics Committee of the UMH and CSIC.

### Animals and treatments

**Figure 1:**
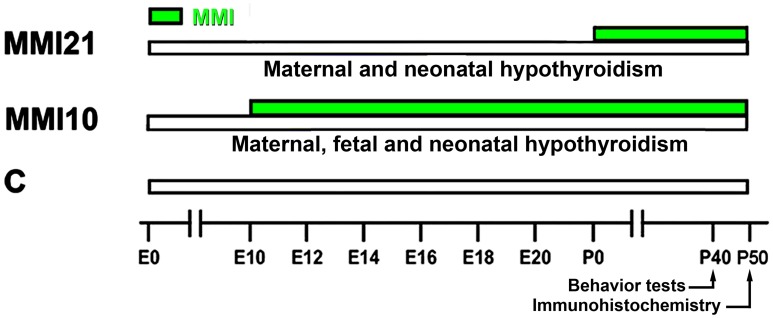
**Experimental groups**. Developmental hypothyroidism was induced by adding 0.02% methimazole (MMI) and 1% KClO4 to the drinking water from E10 (group MMI10) or E21 (group MMI21) until day of sacrifice at P50 (green horizontal bars). Acoustic pre-pulse inhibition and elevated plus-maze tests were performed at P40. Age is indicated at the bottom horizontal bar. E, embryonic day; P, postnatal day.

Wistar rats were housed in temperature-controlled (22–24°C) animal quarters, with automatic light and darkness cycles of 14 and 10 h. Young adult females, weighing 250–300 g, were mated at E0. Hypothyroidism was induced by adding 0.02% methimazole (MMI, Sigma-Aldrich Co., St. Louis, MO) and 1% KClO_4_ to the drinking water starting at E10 (group MMI10; developmental hypothyroidism) or starting at E21 (group MMI21; early postnatal hypothyroidism) until day of sacrifice at P50. The presence of KClO_4_ during gestation and postnatal periods blocks the sodium/iodide symporter (Wyngaarden et al., 1953; Wolff, 1998; Leung et al., 2010). KClO_4_ has a complementary effect to MMI during lactation, since the concentration of MMI in maternal milk has been found to be very low (Azizi et al., 2003). Experimental groups, MMI-treatment periods, age when behavioral test were performed, and age of sacrifice are shown in Figure 1. After weaning, experimental dams anesthetized by inhalation of 1.5–2% isoflurane (Laboratorios Dr. Esteve, S.A., Barcelona, Spain) in O_2_ (0.9 L O_2_/min) were sacrificed by decapitation.

### Determination of total T3 and T4 concentrations in plasma

Blood samples from heart ventricle (5–6 mL) were obtained for 3 groups from dams at day of sacrifice and from their pups at P50 whilst under isoflurane anesthesia. The blood was spun off and the plasma kept at −20°C. Thyroid hormone concentrations were analyzed by radioimmunoassays (RIAs) after extraction and purification of plasma samples, as originally described by Morreale de Escobar et al. (1985). In summary, after adding tracer amounts of [^131^I]-T4 and [^125^I]-T3 to each sample, plasma were extracted and purified using Bio-Rad AG1x2 resin columns (Bio-Rad Laboratories, Hercules, CA). [^131^I]-T4 and [^125^I]-T3 were synthesized in our laboratory using radioactive iodine (Perkin-Elmer, Boston, MA), chloramine T (Sigma-Aldrich, St. Louis, MO) and T3 and 3,5-T2 as substrates (respectively). Recovery of [^131^I]-T4 and [^125^I]-T3 was determined separately in each sample. The sensitivity limits of the RIAs were 2.5 pg T4 and 0.75 pg T3 per tube. Calculations of T4 and T3 concentration (ng/ml) were based on the amount of hormone detected in the RIAs, recovery of the tracers added, and the volume of the extracted plasma sample.

### Conventional histology and immunohistochemistry

We have used the general criteria reported by Amaral and Witter (1995) for the definition of the hippocampal areas and strata. The hippocampal formation is comprised of six areas, which include the DG, the hippocampus proper or CA (containing CA3, CA2, and CA1), subiculum, presubiculum, parasubiculum, and entorhinal cortex. We have focused our study on DG, and on the CA3 and CA1 regions of CA. Using the laminar pattern of VGluT1 labeling, we have subdivided the distal moleculare of the DG into distal-outer and distal-inner layers; the latter being adjacent to the proximal molecular layer and corresponding to the middle molecular layer described by Lynch et al. (1973) (**Figure 7**; asterisk). Each of these three layers (i.e., distal-outer, distal-inner and proximal) covers roughly one third of the thickness of the molecular layer of the DG. In addition, the CA1 stratum radiatum has been divided into two equal sub-layers (proximal and distal), (**Figure 11**).

Pups at P50 were weighed, anesthetized with isoflurane and perfused with 50 mL of saline followed by 200 mL of 4% paraformaldehyde, 0.1M sucrose and 0.002% CaCl_2_ in 0.1M phosphate buffer (PB; 1.4% K_2_HPO_4_ 14 g/L, NaH_2_PO_4_.2H_2_O ~3 g/L to pH 7.3–7.4). The brains were post-fixed by immersion in the same perfusion medium at room temperature for 4 h, and then stored in 0.05% sodium azide in PB at 4°C. Six parallel series of coronal sections, containing the rostromedial portion of the DG, CA and the parietal cortex (−1.8 to −3.8 mm from Bregma), were cut with a Microm HM 650 V vibratome (Thermo Fisher Scientific, Inc., Barcelona, Spain) at 100 μm and stored in 0.05% sodium azide in PB at 4°C. One series was immunostained with anti-mature neurons neuronal nuclei (NeuN) monoclonal Ab (mAb) (1:400; Chemicon International Inc., Temecula, CA). Immunolabeled sections were incubated with biotinylated horse anti-mouse Ab (1:150), Vectastain ABC kit (1:200; both from Vector Laboratories, Inc., Burlingame CA), and 0.05% 3,3′diaminobenzidine (DAB, Sigma-Aldrich Co.). The sections were mounted on gelatinized slides, air dried during 24 h, dehydrated in ethanol, cleared in xylol and coverslipped. The adjacent series was double immunostained for fluorescence, starting with guinea pig anti-VGluT1 antibody (1:5000; Millipore, Temecula, CA) and then rabbit anti-VGAT antibody (1:2000; Synaptic Systems; North Saanich, British Columbia, Canada). All sections were then incubated with goat anti-guinea pig antibody, Alexa Fluor 488 labeled (1:200, Molecular Probes, Invitrogen, Barcelona, Spain), followed with goat biotinylated anti-rabbit antibody (1:200, Vector Laboratories) and NeutrAvidin, Rhodamine Red conjugate (1 mg/ml, Molecular Probes, Invitrogen). Sections were mounted using ProLong Gold (Molecular Probes, Invitrogen), and examined in a Leica TCSL confocal laser fluorescence microscope, with images captured using Leica LCS Lite software.

### Deconvolution and quantitative measurements

**Figure 2:**
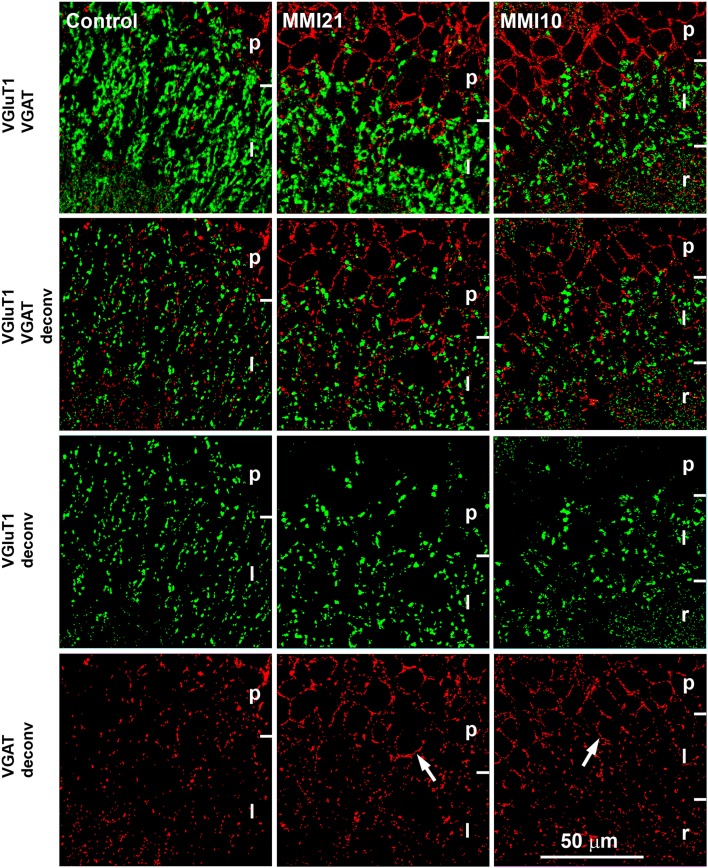
**Deconvoluted confocal images of CA3**. Deconvoluted (deconv) confocal images of VGluT1-ir (green labeling) and VGAT-ir (red labeling) boutons in CA3 strata pyramidale (p), lucidum (l) and radiatum (r) in C and MMI pups. Bouton density and size were analyzed in VGluT1 and VGAT deconv images. In VGAT deconv images, perisomatic inhibitory VGAT-ir boutons in strata pyramidale of MMI pups are shown (arrows). Same scale for all images.

**Figure 3:**
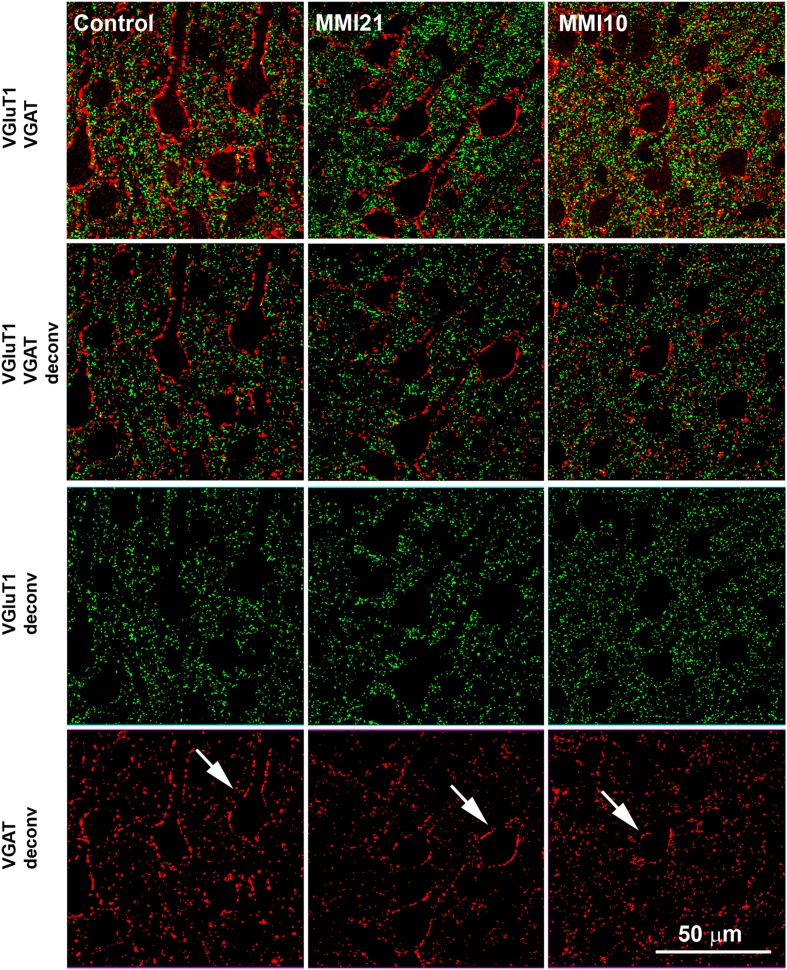
**Deconvoluted confocal images of the somatosensory cortex**. Deconvoluted (deconv) confocal images of VGluT1-ir (green labeling) and VGAT-ir (red labeling) boutons in the layer V of the somatosensory cortex (S1). Bouton density and size was analyzed in VGluT1 and VGAT deconv images. In VGAT deconv images, perisomatic inhibitory VGAT-ir boutons are shown (arrows). Same scale for all images.

Confocal images (1.5 μm-thick and 120 × 120 μm) were deconvoluted and analyzed with ImageJ software (mask set from 3 to 8 pixels) (Figures 2, 3). VGluT1-ir and VGAT-ir boutons were counted in 8 deconvoluted images picked at random, whilst avoiding overlapping between layers in DG, CA3, CA1 and the primary somatosensory cortex (5 layers per cortical region were analyzed, except for the somatosensory cortex that included subcortical white matter). Four pups were studied per experimental condition, resulting in a total of 40 images per region and pup (48 in the somatosensory cortex). VGluT1-ir and VGAT-ir bouton density (per 10^4^ μm^2^) was obtained $$$(panels A and B from **Figures 8**, **10**, **12**, **14**, and Supplementary Tables 1–4), and bouton size measured $$$(panels F and G from **Figures 8**, **10**, **12**, **14**, and Supplementary Tables 1–4) from 1344 confocal images per experimental group; 672 green and 672 red. The percentage of immunolabeled boutons among layers $$$(panels C and D from **Figures 8**, **10**, **12**, **14**, and Supplementary Tables 1–4) was obtained according to the layer thickness. The percentage of VGAT-ir boutons within each layer $$$(panel E from **Figures 8**, **10**, **12**, **14**, and Supplementary Tables 1–4) was calculated as the ratio between the number of VGAT-ir boutons and the total VGluT1-ir and VGAT-ir boutons in each layer. This value reflects changes in bouton density rather than changes in percentages between layers. The bouton size was measured by counting the number of labeled pixels using Image J, and is an estimation of the area occupied by synaptic vesicles inside the bouton.

### Prepulse inhibition of the acoustic startle response

Prepulse inhibition of the acoustic startle response, i.e., the reduction of the response by a weak prepulse preceding the startle pulse, was used as a measure of sensorimotor gating mechanisms (Graham, 1975; Norris and Blumenthal, 1996; Koch, 1999; Plappert et al., 2004). Pups were placed in soundproof chambers equipped with loudspeakers controlled by the STARTLE software and interface system (Panlab, Barcelona, Spain). Pup movement inside a Plexiglas cylinder was measured by a piezoelectric accelerometer and converted into a digital signal. Pups were submitted to the prepulse inhibition paradigm using a previously described protocol (Paylor and Crawley, 1997). Rats were acclimatized three days prior to test sessions by placing them each day in the apparatus for 5 min without background noise. Tests sessions began with a habituation phase using a constant 65 dB background noise with the rat undisturbed for 10 min. After habituation, each rat was presented 80 trials in pseudorandom order over a 37-min test interval. The trials included 120 dB acoustic startle stimulus for 40 ms (which does not cause cochlear damage; Lawner et al., 1997; Kujawa and Liberman, 2009), 3 × 20 ms prepulse stimulus (74, 82, and 90 dB) and 3 × 20 ms prepulse (100 ms before the onset) as well as startle stimulus trials. Finally, trials where no stimulus was present were used to measure baseline movements. The average inter-trial interval was 15 s and the maximum startle amplitude was recorded during a 100 ms sampling window. The recording window was established at 100 ms to avoid registering any movement not related with the startle (for example the movement of the animal inside the restrainer during the inter-trial interval that could possibly be even higher than the startling response). The mean percentage of prepulse inhibition, achieved with each intensity and mean startle amplitude during pulse only trials, was analyzed. The ratio of startle response was calculated as the sum of startle response on acoustic prepulse and startle stimulus to the startle response. The prepulse inhibition percentage was 100 × (1 –startle response ratio).

### Elevated plus maze

The elevated plus-maze consisted of a plus-shaped apparatus elevated 50 cm above the floor with two open and two enclosed arms with an open roof. The junction of the four arms forms a square central platform (5 × 5 cm). The open space of the open arms provides an anxiogenic stimulus and the test evaluates anxiety by measuring the time spent in the open arms; less anxiety results in an increased proportion of time spent in the open arms, and/or an increase in the proportion of entries into the open arms (Pellow et al., 1985; Lister, 1987; Engin and Treit, 2007). The pup started the test placed in the center of the apparatus facing one of the enclosed arms and it was allowed to freely explore the maze for 5 min. During this period, the time spent in open arms (as percentages of total test time) and the number of entries from open-arms to closed-arms (and vice versa) was recorded. Arm entry was registered when all four paws passed into an arm.

### Statistical analysis

For statistical analysis we used the SYSTAT software (Systat Software, Inc., Chicago, IL). Density, frequency distributions and size of VGluT1 and VGAT-ir boutons were analyzed using Two-Way ANOVA followed by either Tukey's (equal variances) or Games-Howell's (unequal variances) tests to identify significant differences (*P* ≤ 0.05) between means among strata and experimental groups. One-way ANOVA followed either by Tukey's test or by the Student-Newman-Keuls method were used to analyze the concentrations of thyroid hormones in plasma at P50 or the behavior of the pups at P40, respectively.

## Results

### Thyroid hormone levels

**Figure 4:**
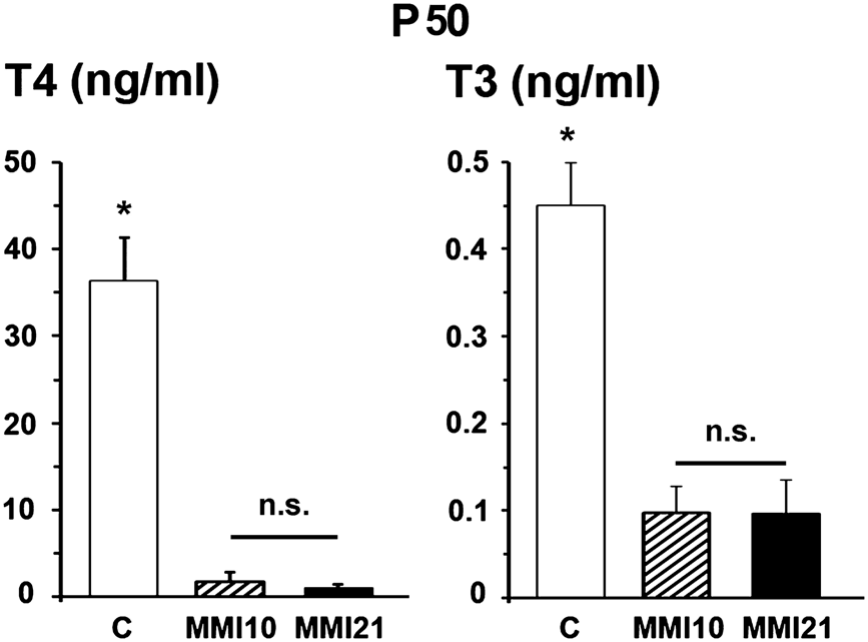
**Circulating total thyroid hormone levels**. Bar diagrams showing total T4 and T3 plasmatic levels of C and MMI pups at P50. Asterisks show significant differences (*P* ≤ 0.001) between C and MMI pups. n.s., indicates not significant differences.

At P50, total plasmatic T4 levels of both MMI10 (1.86 ng/ml) and MMI21 (1.08 ng/ml) pups were significantly lower (*P* < 0.001) than those of C (36.29 ng/ml) pups. Also, the total plasmatic T3 levels of MMI10 (0.10 ng/ml) and MMI21 (0.10 ng/ml) were significantly lower (*P* < 0.001) than those of C (0.45 ng/ml) pups. No significant differences were found in circulating total T4 and T3 levels between MMI10 and MMI21 pups (Figure 4).

### Immunolabeling of NeuN, VGluT1 and VGAT in DG and CA

**Figure 5:**
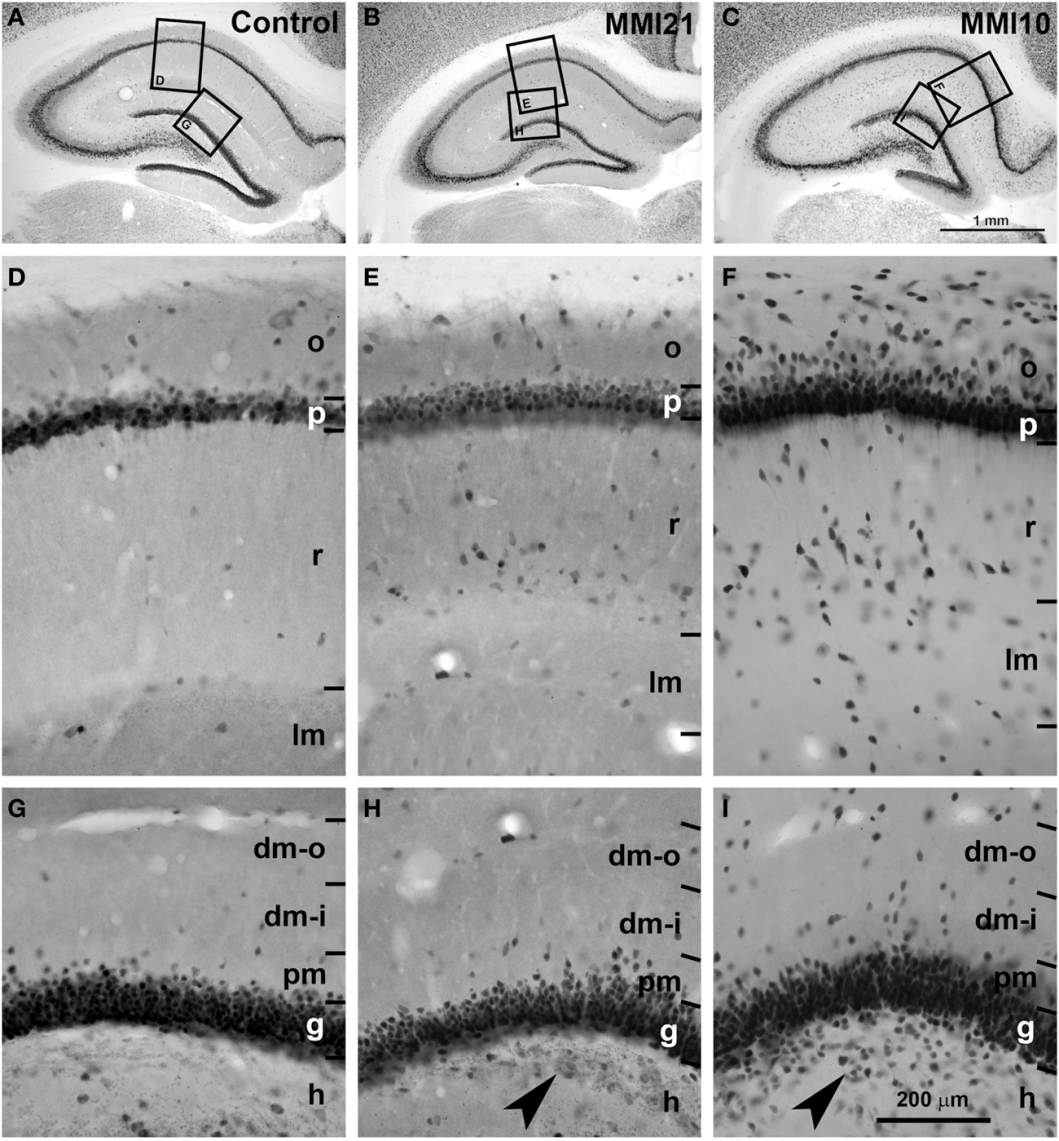
**Low magnification photomicrographs of NeuN-immunostained coronal sections of the hippocampus**. Low magnification photomicrographs of coronal sections of the hippocampus showing NeuN-ir neurons in C and MMI pups at P50. Details of NeuN-ir neurons in CA1 **(D–F)** and DG **(G–I)** of C and MMI pups. The border between the CA1 pyramidal (p) and DG granular (g) and adjacent layers are more blurred in MMI pups than in C pups **(E,F,H,I)**. Note the increased number of heterotopic neurons in strata oriens (o) and radiatum (r) in CA1 **(E,F)**, and in distal (dm-o and dm-i) and proximal (pm) molecular layers, and hilus (h; arrowheads in **H,I**) in DG of MMI pups compared to controls. Boxes in **(A–C)** show enlarged images in **(D–I)**. dm-o, Distal-outer molecular; dm-i, distal-inner molecular. Same scale for **(A–C)**, and for **(D–I)**.

Low magnification of NeuN-immunostained sections (Figures 5A–C) showed abnormal laminar organization of the hippocampus in MMI (MMI10 and MMI21) pups (Figures 5B,C). In CA1, heterotopic neurons were observed in strata oriens and radiatum of MMI21 pups (Figure 5E), and in strata oriens, radiatum and lacunosum-moleculare of MMI10 pups (Figure 5F). In DG, heterotopic neurons were observed in the proximal molecular layer of MMI21 pups (Figure 5H), and in distal and proximal layers of MMI10 pups (Figure 5I). Heterotopic NeuN-ir neurons were also observed in the hilus of the DG of MMI pups (Figures 5H,I, arrowheads). In MMI pups, the borders of the CA1 pyramidal and the DG granular layers with the adjacent oriens and proximal molecular layers, respectively, were more blurred than in C pups (compare Figures 5E,F,H,I with Figues 5D,G).

**Figure 6:**
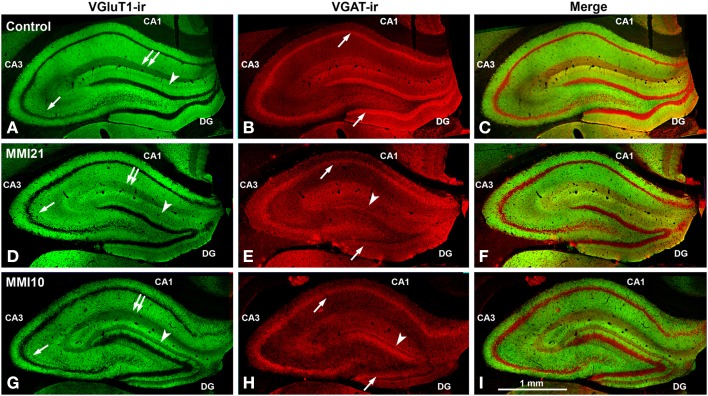
**Low magnification confocal images showing abnormal immunolabeling in MMI pups**. Collages of confocal photomicrographs showing VGluT1-ir (green labeling; **A,D,G**), VGAT-ir (red labeling; **B,E,H**) and merged images **(C,F,I)** in the hippocampus of C **(A–C)**, MMI21 **(D–F)** and MMI10 **(G–I)** pups at P50. Note the decreased VGluT1-ir in DG distal-inner molecular layer (arrowhead in **A,D,G**), in CA3 strata lucidum (arrow in **A,D,G**) and radiatum, and in CA1 stratum lacunosum-moleculare (double arrows in **A,D,G**) in MMI compared to C pups. These data show that the basic trisynaptic loop in MMI pups is abnormal. A narrow band of increased VGAT-ir boutons located at the border between the DG distal and proximal molecular layers of MMI pups is indicated (arrow heads in E,H). The borders of the principal cell layer, defined by VGAT immunolabeling) are more blurred in MMI pups compared to controls (arrows in **B,E,H**). Same scale for **(A–I)**.

**Figure 7:**
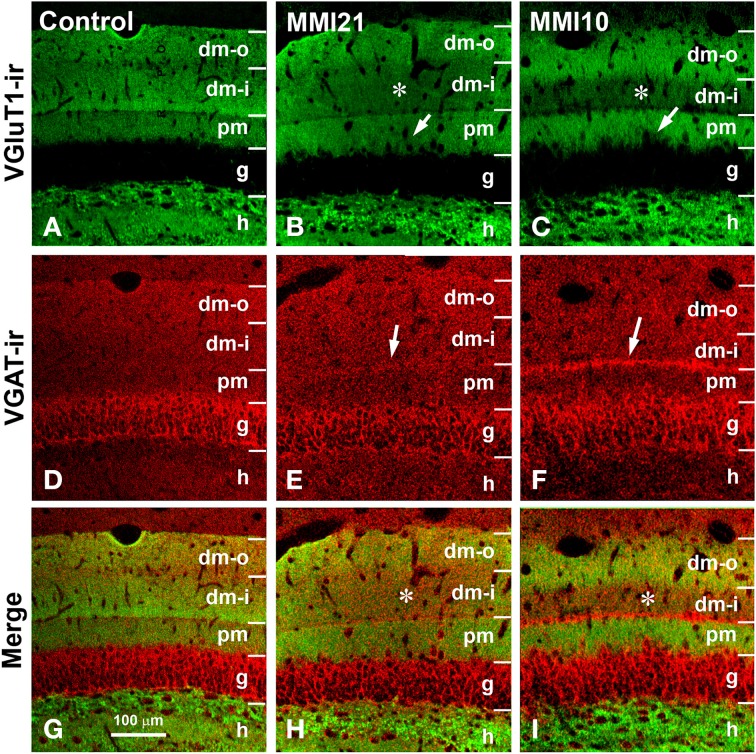
**Confocal immunolabeling in DG of C and MMI pups**. Confocal photomicrographs showing VGluT1-ir (green labeling; **A–C**), VGAT-ir (red labeling; **D–F**), and merged images **(G–I)** in DG of C **(A,D,G)**, MMI21 **(B,E,H)** and MMI10 **(C,F,I)** pups at P50. The border between the granular (g) and proximal molecular (pm) layers is blurred in MMI pups (arrows in **B,C**). Note the decreased density of VGluT1-ir boutons in the distal-inner molecular layer (dm-i) (asterisks in **B,C,H,I**). A narrow band of VGAT-ir boutons between the distal-inner (dm-i) and proximal (pm) molecular layers can be seen in MMI pups (arrows in **E,F**). dm-o, Distal-outer molecular; h, hilus. Same scale for **(A–I)**.

Low power confocal micrographs (Figures 6A–I) resulting from the overlay of 4 consecutive optical sections (covering 6 μm depth) showed that the distribution of both VGluT1-ir and VGAT-ir boutons in DG, CA3, and CA1 of MMI pups was abnormal and reflected in an alteration of the laminar distribution of excitatory and inhibitory input (see also Figures 7A–I, 9A–I, **11A–I**). In the DG (Figures 7A–I), the most prominent findings were an additional band of VGAT-ir boutons at the border between proximal and distal-inner molecular layers (arrowheads in Figures 6E,H, and arrows in Figures 7E,F), and a very low VGluT1-ir bouton density in the distal-inner molecular layer of MMI pups (arrowheads in Figures 6D,G, and asterisks in Figures 7B,C). In MMI pups, the VGluT1-ir bouton percentage decreased in strata oriens, lucidum and radiatum of CA3 (arrows in Figures 6D,G, and arrowheads in Figures 9A–C) and in stratum lacunosum-moleculare of CA1 (double arrows in Figures 6D,G, **11B,C**) in comparison to C pups. In particular, the area occupied by VGluT1-ir boutons (mossy boutons) in strata oriens and lucidum of CA3 was less than in C pups (arrows in Figures 6D,G, and arrowheads in Figures 9A–C). In CA1 (**Figures 11A–I**), heterotopic VGluT1-ir neurons were present in the strata oriens and radiatum of MMI pups, being most numerous in MM10 pups (**Figures 11B,C**; arrows). In addition, MMI pups showed blurred borders between the different layers, especially in the CA1 pyramidal cell layer (**Figure 11C**).

### Distribution of VGluT1-ir and VGAT-ir boutons in DG

**Figure 8:**
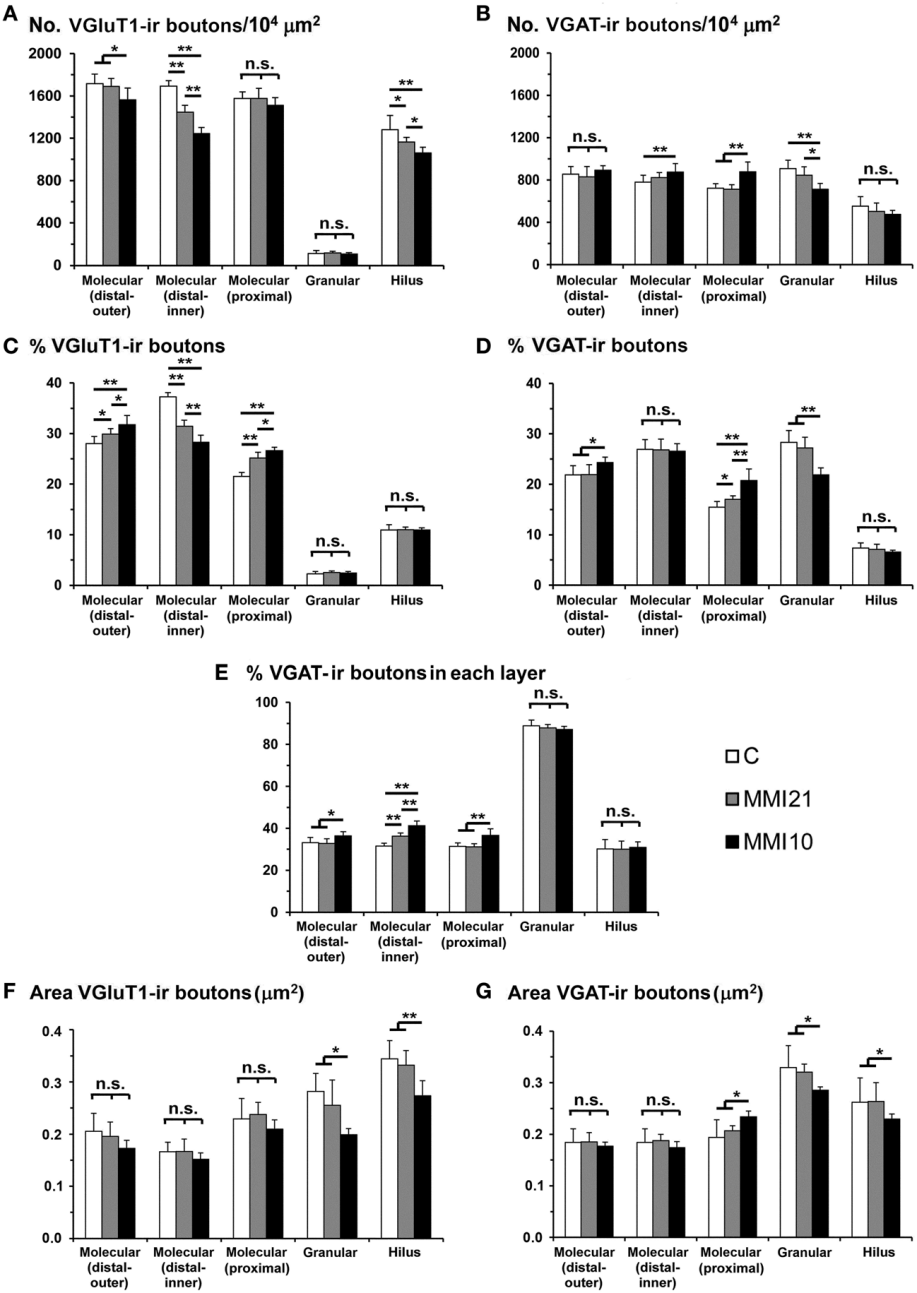
**VGluT1-ir and VGAT-ir bouton distribution in DG of C and MMI pups**. Histograms showing the VGluT1-ir and VGAT-ir bouton distribution in DG of C and MMI pups. Note the deceased VGlut1-ir bouton density and percentage in the distal-inner molecular layer of MMI compared to C pups **(A,C)**. The VGAT-ir bouton density and percentage increased in the proximal molecular layer and decreased in the granular layer of MMI10 pups, and increased in the proximal molecular layer of MMI21 pups **(B,D)**. The VGAT-ir bouton percentage in each layer increased in the MMI10 distal (outer and inner) and proximal molecular and granular layers, and in the MMI21 distal-inner molecular layer **(E)**. The VGluT1-ir and VGAT-ir bouton area was smaller in the MMI10 granular layer and hilus **(F,G)**. n.s. indicates not significant differences; (^*^) and (^**^) indicate significant differences, *P* ≤ 0.05 and *P* ≤ 0.001, respectively.

VGluT1-ir bouton density and percentage in MMI pups were significantly less in the distal-inner molecular layer (Figures 8A,C; Supplementary Table 1). In addition, MMI VGluT1-ir bouton percentage significantly increased in the distal-outer and proximal molecular layers (Figure 8C; Supplementary Table 1). In all groups, the lowest VGAT-ir bouton density and percentage was found in the hilus. VGAT-ir bouton density significantly decreased in the granular layer of MMI pups, but increased in the distal-inner and proximal molecular layers of MMI10 pups (Figures 8B,D; Supplementary Table 1). MMI VGAT-ir bouton percentage significantly increased in the distal-inner molecular layer, and in MMI10 pups increased in the distal-outer molecular layer and decreased in the granular layer (Figure 8D; Supplementary Table 1). In all groups, the VGAT-ir bouton percentage in each layer (reflecting the VGAT-ir to VGluT1-ir bouton density ratio) was largest in the granular layer (Figure 8E; Supplementary Table 1). The VGAT-ir bouton percentage showed an increase in the MMI distal-inner molecular layer as well as the MMI10 distal-outer and proximal molecular layers (Figure 8E; Supplementary Table 1).

**Figure 9:**
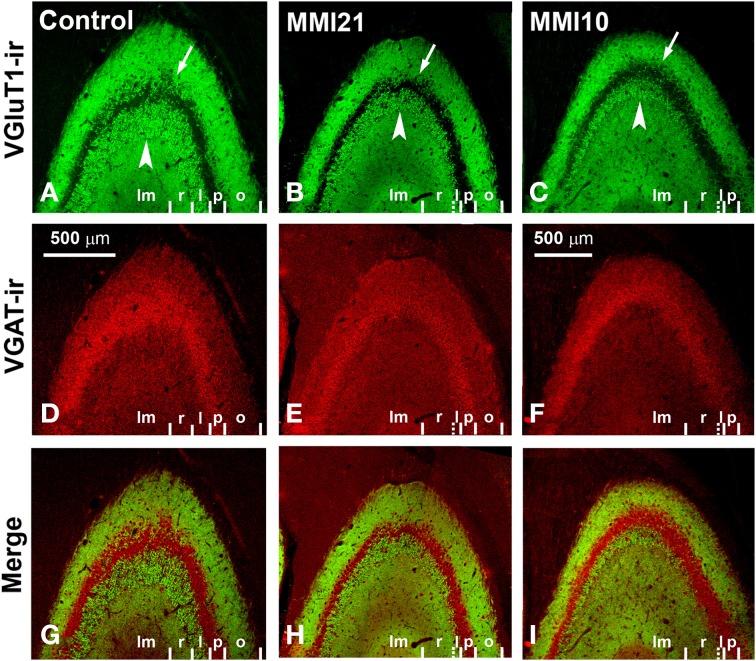
**Confocal immunolabeling in CA3 of C and MMI pups**. Confocal photomicrographs showing VGluT1-ir (green labeling; **A–C**), VGAT-ir (red labeling; **D–F**) and merged images **(G–I)** in CA3 of C **(A,D,G)**, MMI21 **(B,E,H)** and MMI10 **(C,F,I)** pups at P50. Note that the area occupied by VGluT1-ir mossy boutons in the strata oriens (o; arrows) and lucidum (l; arrowheads) of CA3 is less in MMI21 **(B)** and MMI10 **(C)** pups than in controls **(A)**. The border between the strata lucidum (l) and radiatum (r) are marked in dashed lines in **(B,C)**, owing to the low density of labeled boutons. p, Pyramidale; lm, lacunosum-moleculare. Same scale for **(A–I)**.

In all groups, VGluT1-ir bouton area was largest in hilus and lowest in the distal-inner molecular layer. VGluT1-ir bouton area decreased in the MMI10 granular layer and hilus (Figure 8F; Supplementary Table 1). In all groups, VGAT-ir bouton area was largest in the granular layer and lowest in the distal molecular layer. MMI10 VGAT-ir bouton area increased in the proximal molecular layer and decreased in the granular layer and hilus (Figure 8F; Supplementary Table 1).

### Distribution of VGluT1-ir and VGAT-ir boutons in CA3

**Figure 10:**
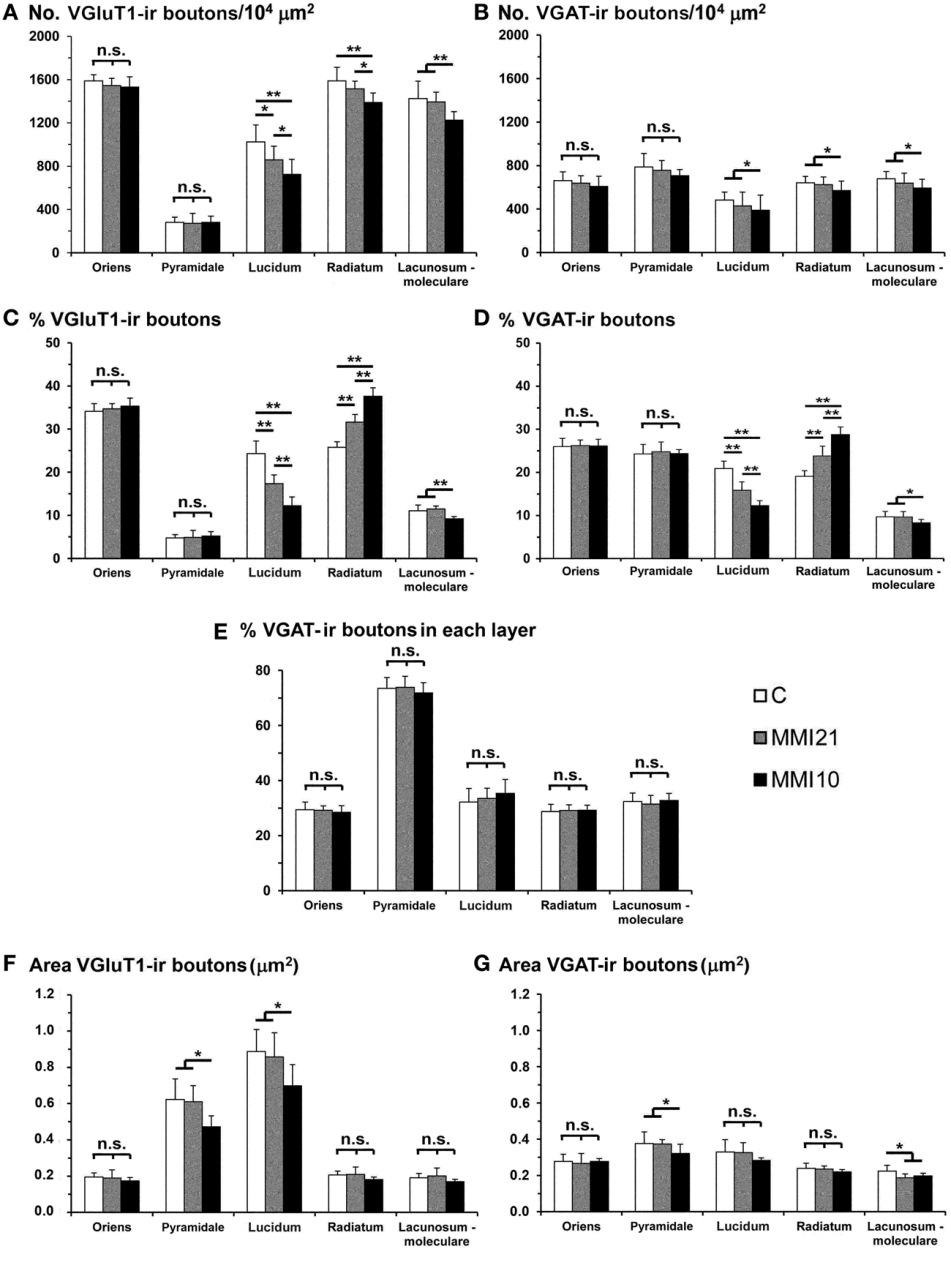
**VGluT1-ir and VGAT-ir bouton distribution in CA3 of C and MMI pups**. Histograms showing the VGluT1-ir and VGAT-ir bouton distribution in CA3 of C and MMI pups. The VGluT1-ir and VGAT-ir bouton density and percentage decreased in the MMI stratum lucidum, and bouton density increased in the stratum radiatum **(A–D)**. Despite of the differences found in the VGluT1-ir and VGAT-ir bouton density and percentage in MMI pups, the VGAT-ir bouton percentage in each stratum was similar in all groups **(E)**. The VGluT1-ir bouton area decreased in MMI10 strata pyramidale and lucidum **(F)**. The VGAT-ir bouton area decreased in MMI10 strata pyramidale and lacunosum-moleculare, and in MMI21 stratum lacunosum-moleculare **(G)**. n.s. indicates not significant differences; (^*^) and (^**^) indicate significant differences, *P* ≤ 0.05 and *P* ≤ 0.001, respectively.

MMI VGluT1-ir bouton density decreased in the strata lucidum and radiatum, and in MMI10 stratum lacunosum-moleculare. In MMI pups, VGluT1-ir bouton percentage decreased in stratum lucidum and increased in the stratum radiatum, and decreased in MMI10 stratum lacunosum-moleculare (Figure 10C; Supplementary Table 2). In all groups, VGAT-ir bouton density was largest in the stratum pyramidale and smallest in the lucidum, while it decreased in MMI10 strata lucidum, radiatum and lacunosum-moleculare (Figure 10B; Supplementary Table 2). MMI VGAT-ir bouton percentage decreased in stratum lucidum and increased in stratum radiatum, and decreased in MMI10 stratum lacunosum-moleculare (Figure 10D; Supplementary Table 2). In all groups, the VGAT-ir bouton percentage in each layer (reflecting the VGAT-ir to VGluT1-ir bouton density ratio) was largest in stratum pyramidale. No significant differences were found between C and MMI VGAT-ir bouton percentages (Figure 10E).

**Figure 11:**
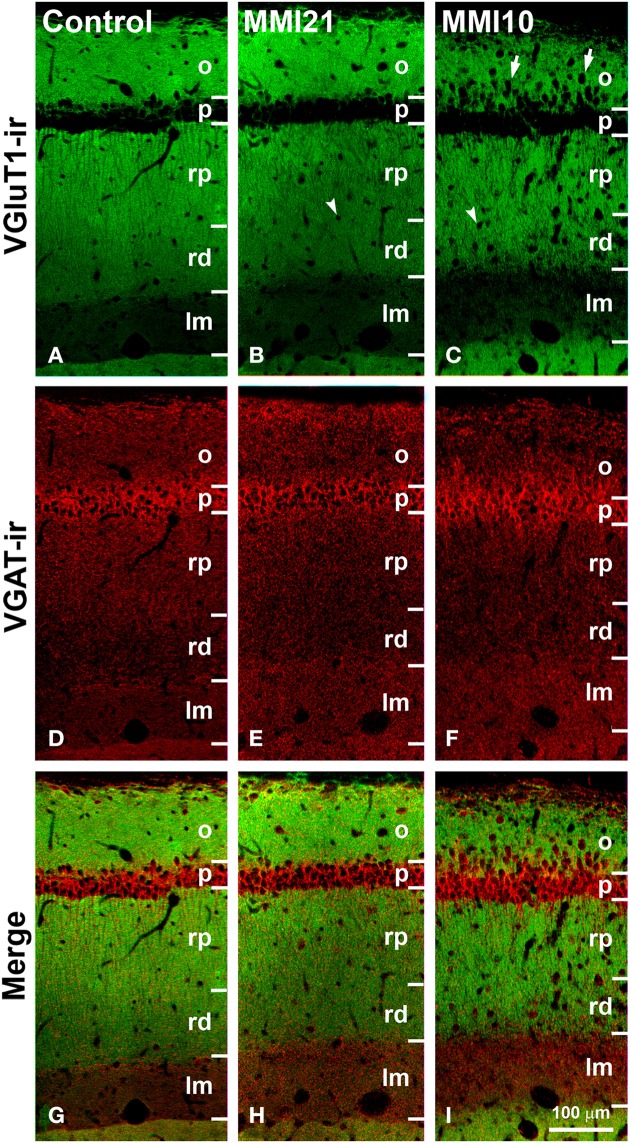
**Confocal immunolabeling in CA1 of C and MMI pups**. Confocal photomicrographs showing VGluT1-ir (green labeling; **A–C**), VGAT-ir (red labeling; **D–F**) and merged images **(G–I)** in CA1 of C **(A,D,G)**, MMI21 **(B,E,H)** and MMI10 **(C,F,I)** pups at P50. Note the increased thickness of the stratum lacunosum-moleculare (lm) in MMI pups (compare **B,C** with **A**), and the blurred border between the strata pyramidale (p) and oriens (o) in MMI10 pups (arrows in C). An increased number of cells can be seen in strata oriens (o) and radiatum (rp and rd) of MMI pups (arrows and arrowheads in B and C point to immunonegative cell somata), compared to controls **(A)**. Some of these ectopic cells resemble pyramidal neurons (arrowheads in **B,C**; see also Figures 5E,F). rp, Proximal radiatum; rd, distal radiatum. Same scale for **(A–I)**.

In all groups, VGluT1-ir bouton area was largest in stratum lucidum and smallest in stratum pyramidale (Figure 10F; Supplementary Table 2). MMI10 VGluT1-ir bouton area decreased in strata pyramidale and lucidum (Figure 10F; Supplementary Table 2). In all groups, VGAT-ir bouton area was greater in stratum pyramidale (Figure 10G; Supplementary Table 2). MMI10 VGAT-ir bouton area decreased in strata pyramidale and lacunosum-moleculare (Figure 10G; Supplementary Table 2).

### Distribution of VGluT1-ir and VGAT-ir boutons in CA1

**Figure 12:**
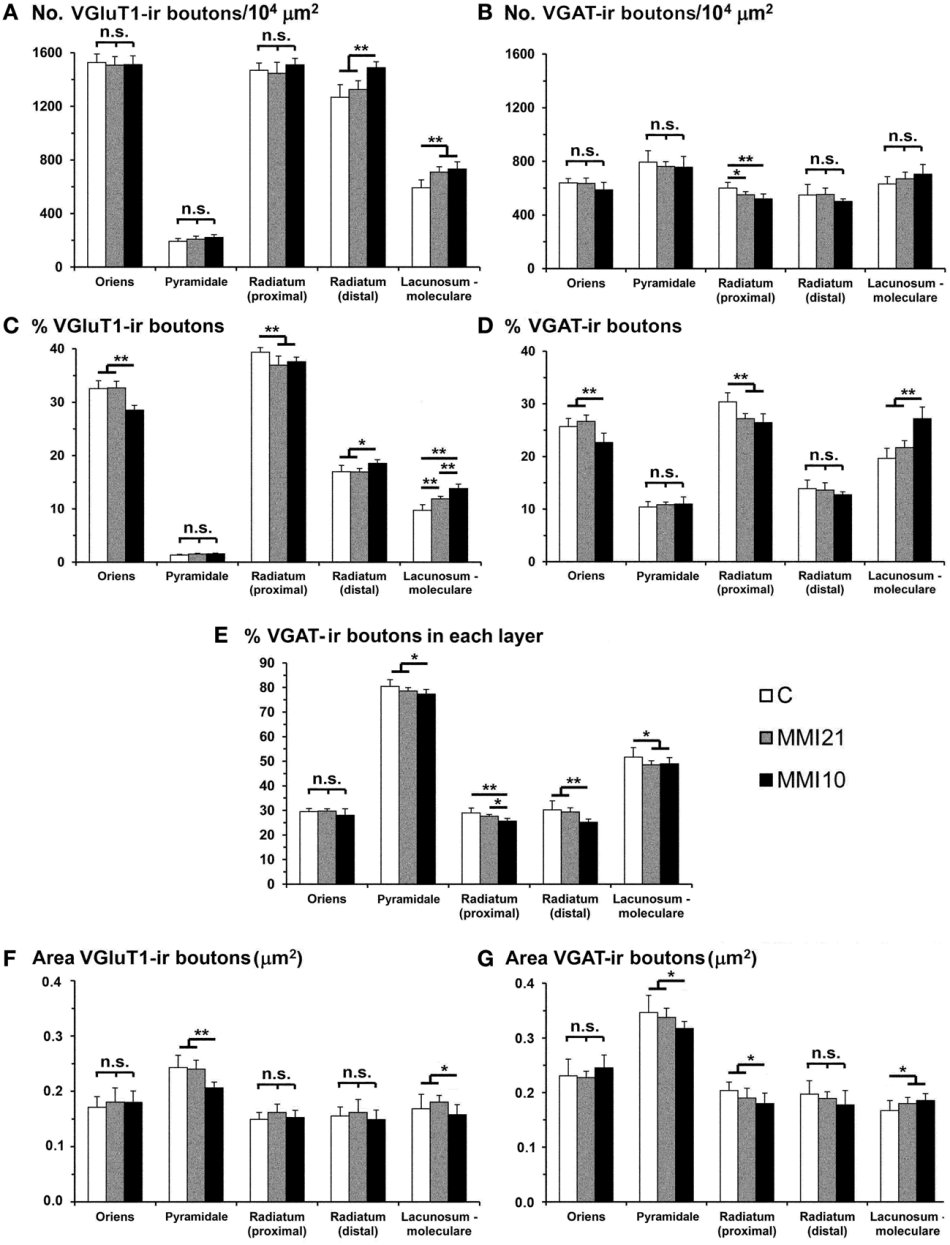
**VGluT1-ir and VGAT-ir bouton distribution in CA1 of C and MMI pups**. Histograms showing the VGluT1-ir and VGAT-ir bouton distribution in CA1 of C and MMI pups. The VGluT1-ir and VGAT-ir bouton density increased in MMI stratum lacunosum-moleculare and decreased in proximal radiatum **(A,B)**. Significant differences between C and MMI VGluT1-ir bouton percentage were found in all the strata **(C)**. VGAT-ir bouton percentage decreased in MMI10 stratum oriens, increased in MMI10 stratum lacunosum-moleculare, and decreased in MMI proximal radiatum **(D)**. The VGAT-ir bouton percentage in each stratum decreased in MMI10 strata pyramidale and radiatum, and in MMI lacunossum-moleculare **(E)**. VGluT1-ir bouton area decreased in MMI10 strata pyramidale and lacunosum-moleculare **(F)** and VGAT-ir bouton area decreased in MMI10 strata pyramidale and proximal radiatum, and increased in MMI stratum lacunosum-moleculare **(G)**. n.s. indicates not significant differences; (^*^) and (^**^) indicate significant differences, *P* ≤ 0.05 and *P* ≤ 0.001, respectively.

VGluT1-ir bouton density and percentage increased in MMI stratum lacunosum-moleculare and in MMI10 distal radiatum, while VGluT1-ir bouton percentage decreased in MMI stratum proximal radiatum and in MMI10 oriens (Figures 12A,C; Supplementary Table 3). In all groups, VGAT-ir bouton density was largest in the stratum pyramidale. VGAT-ir bouton percentage decreased in MMI stratum proximal radiatum and in MMI10 stratum oriens, but increased in MMI10 lacunosum-moleculare (Figures 12B,D; Supplementary Table 3). In all groups, the VGAT-ir bouton percentage in each layer (reflecting the VGAT-ir to VGluT1-ir bouton density ratio) was largest in strata pyramidale and lacunosum-moleculare (Figure 12E; Supplementary Table 3). VGAT-ir bouton percentage decreased in MMI stratum lacunosum-moleculare and proximal radiatum, as well as in MMI10 strata pyramidale and distal radiatum (Figure 12E; Supplementary Table 3).

In all groups, VGluT1-ir bouton area was largest in the stratum pyramidale, while MMI10 VGluT1-ir bouton area decreased in strata pyramidale and lacunosum-moleculare (Figure 12F; Supplementary Table 3). In all groups, VGAT-ir bouton area was largest in stratum pyramidale. MMI VGAT-ir bouton area increased in the stratum lacunosum-moleculare and decreased in MMI10 strata pyramidale and proximal radiatum (Figure 12G; Supplementary Table 3).

### Distribution of VGluT1-ir and VGAT-ir boutons in the somatosensory cortex

**Figure 13:**
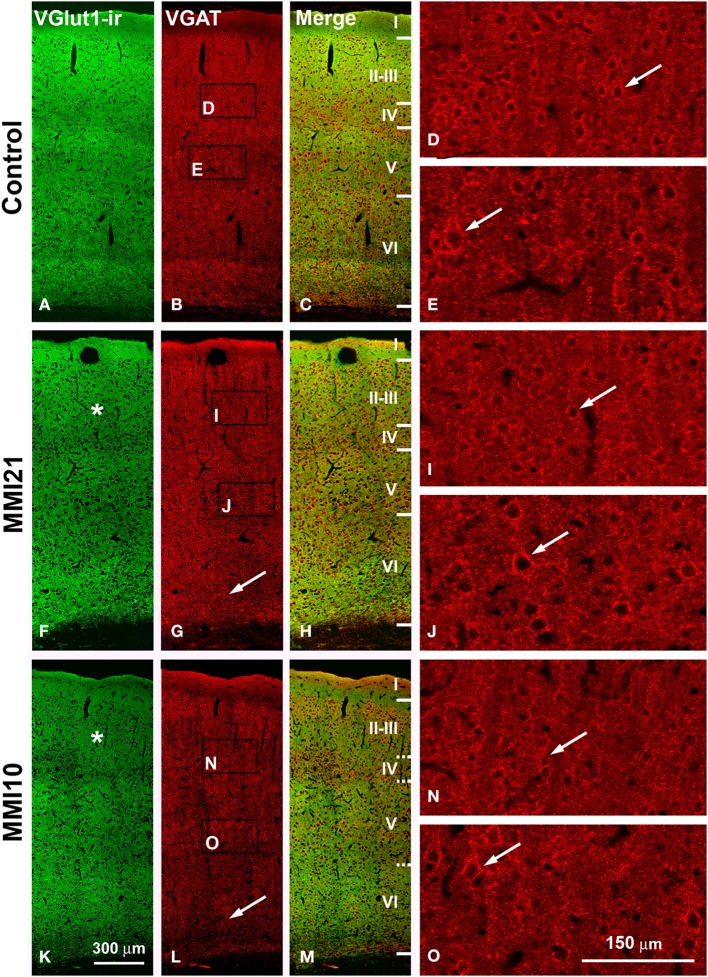
**Confocal immunolabeling in the somatosensory cortex of C and MMI pups**. Confocal photomicrographs showing VGluT1-ir (green labeling; **A,F,K**), VGAT-ir (red labeling; **B,G,L**) and merged images **(C,H,M)** in the somatosensory cortex of C **(A–E)**, MMI21 **(F–J)** and MMI10 **(K–O)** pups at P50. Asterisks (^*^) point to supragranular VGluT1-ir labeling in MMI **(F,K)** pups. Perisomatic VGAT-ir boutons are indicated by arrows. Boxes in **(B,G,L)** show the location of the corresponding enlarged figures. Note the decreased density and smaller size of perisomatic VGAT-ir boutons (arrows) in layers II-III and V in MMI10 pups (compare **N,O** with **D,E,I,J**). Same scale for **(A–C, F–G, K–M)** and for **(D,E,I,J,N,O)**.

Low power confocal micrographs resulting from the overlay of 4 consecutive sections (covering 6 μm depth) showed that the distribution of VGluT1-ir and VGAT-ir boutons in the somatosensory cortex of MMI pups was abnormal. The most prominent findings in MMI pups were a decrease of VGluT1-ir bouton density in layer II–III (asterisks in Figures 13F,K) and of VGAT-ir bouton density in layer VI (arrows in Figures 13G,L). In MMI10 pups, decreased VGluT1-ir bouton density in layer IV and VGAT-ir bouton density in layers II–V were also observed (Figures 13K,L). In MMI pups, the borders between layers were blurred (Figure 13; compare H and M with C panels). The VGAT-ir bouton density in layers II-VI in MMI10 pups reflects a significant reduction in number and complexity of perisomatic VGAT-ir boutons (Figure 13; compare N and O with D, E, I, and J panels).

**Figure 14:**
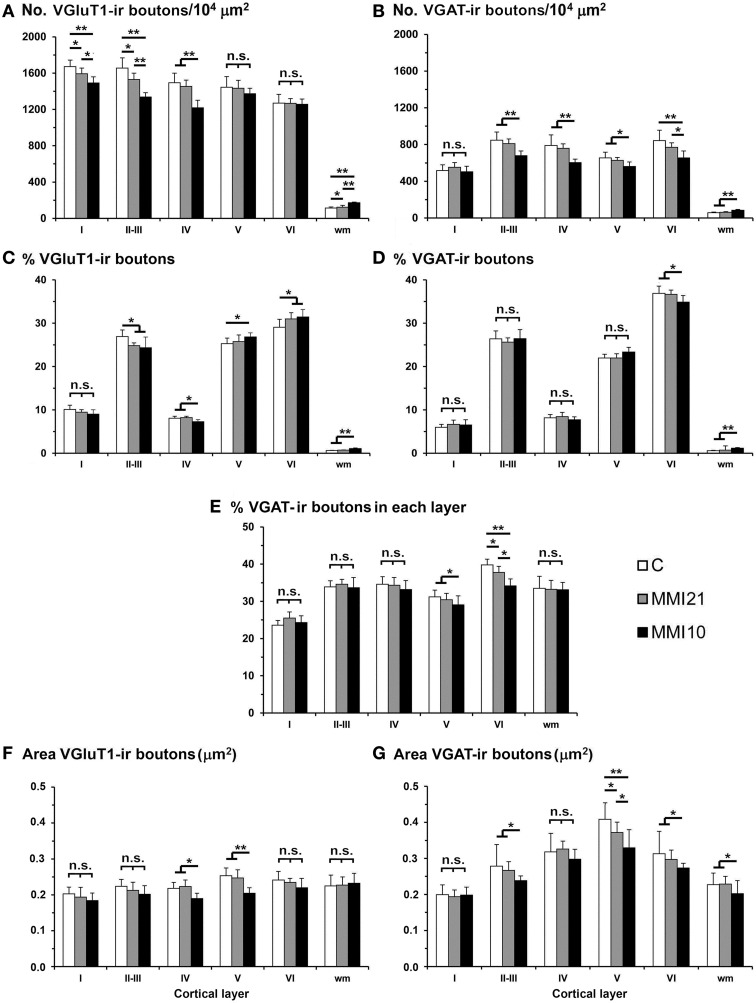
**VGluT1-ir and VGAT-ir bouton distribution in the somatosensory cortex of C and MMI pups**. Histograms showing the VGluT1-ir and VGAT-ir bouton distribution in the somatosensory cortex of C and MMI pups. The VGluT1-ir bouton density decreased in layers I-III of MMI pups and layer IV of MMI10 pups **(A)**, and the VGAT-ir bouton density decreased in layer VI of MMI pups and layers II-V of MMI10 pups **(B)**. Significant differences between C and MMI VGluT1-ir bouton percentage were found in II-III and VI; MMI10 VGluT1-ir bouton percentage also was different in layers IV and V **(C)**. The VGAT-ir bouton percentage decreased in layer VI of MMI10 pups **(D)**. The VGAT-ir bouton density in each layer decreased in layer VI of MMI pups and in layer V of MMI10 pups **(E)**. The VGluT1-ir bouton area decreased in layers IV and V of MMI10 pups **(F)** and VGAT-ir bouton area decreased in layer V of MMI pups and layers II-III and VI of MMI10 pups **(G)**. n.s. indicates not significant differences; (^*^) and (^**^) indicate significant differences, *P* ≤ 0.05 and *P* ≤ 0.001, respectively.

VGluT1-ir bouton density and percentage decreased in MMI layer II-III and in MMI10 layer IV (Figures 14A,C; Supplementary Table 4). In addition, VGluT1-ir bouton density increased in MMI10 layers V and VI, and in the subcortical white matter of MMI pups. In all groups, VGAT-ir bouton density and percentage was largest in layers II–III and VI. VGAT-ir bouton density decreased in MMI layer VI, and in MMI10 layers II–III, IV, and VI (Figures 14B,D; Supplementary Table 4). In all groups, the VGAT-ir bouton percentage in each layer (reflecting the VGAT-ir to VGluT1-ir bouton density ratio) was was largest in layer V. However, VGAT-ir bouton percentage decreased in MMI layer VI and in MMI10 layer V (Figure 14E; Supplementary Table 4).

In MMI10 pups, VGluT1-ir bouton area decreased in layers IV and V (Figure 14F; Supplementary Table 4). In all groups, VGAT-ir bouton area was largest in layer V. VGAT-ir bouton area decreased in MMI layer V and in MMI10 layers II–III and VI (Figure 14G; Supplementary Table 4).

### Prepulse inhibition of the acoustic startle response

**Figure 15:**
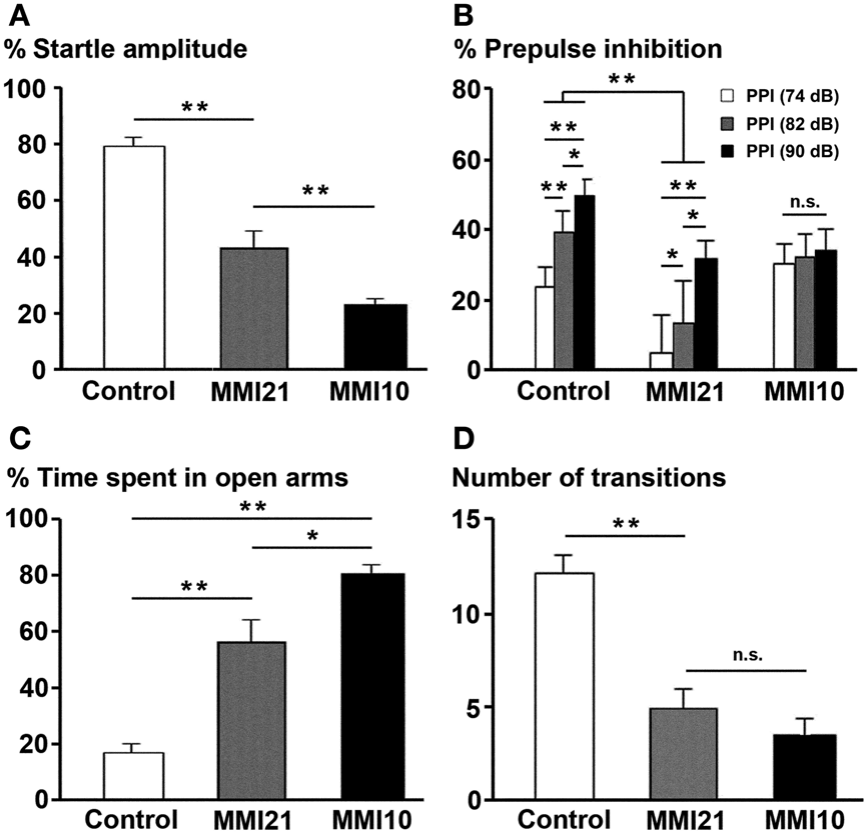
**Prepulse inhibition of the acoustic startle response and elevated plus maze tests**. Bar diagram showing the the startle amplitude **(A)**, percentage of prepulse inhibition **(B)**, and time spent in open arms **(C)** of C and MMI pups at P40. In MMI pups, both the acoustic startle response amplitude and the percentage of prepulse inhibition at 74, 82 and 90 dB prepulses decreased, while the percentage of time in the open arms of the elevated plus-maze increased and the number of transitions decreased **(D)**. (^*^) and (^**^) indicate significant differences, *P* ≤ 0.05 and *P* ≤ 0.001, respectively.

At P40, the percentage of acoustic startle response amplitude was significantly reduced in MMI pups (*P* < 0.001). It was 23.3% in MMI10, 43.0% in MMI21 and 79.0% in C pups (Figure 15A). The presentation of 74, 82, and 90 dB prepulse stimuli revealed a significant difference between C (30.0% PPI at 74 dB; 44.1% PPI at 82 dB and 54.5% PPI at 90 dB) and MMI21 (5.9, 18.0 and 32.1% PPI, respectively) pups at all prepulse values (*P* < 0.001). In addition, a significant prepulse sound escalation was found in C and MMI21 pups (*P* < 0.05) (Figure 15B). In contrast, MMI10 pups did not respond differently to the varying sound intensities (30.2% PPI at 74 dB; 32.0% PPI at 82 dB and 34.1% PPI at 90 dB) (Figure 15B). These data show a severe pre-attention deficit in MMI21 pups, with MMI10 pups showing a very low response to auditory stimuli most likely due to severely impaired hearing (see Discussion).

### Elevated plus maze

When tested in the elevated plus maze, MMI pups showed a largest preference for the open arms (57.0% time spent in open arms in MMI21 and 81.1% in MMI10 pups) compared to C pups (17.1%; *P* < 0.001; Figure 15C). In addition, it was not uncommon for MMI10 pups to fall from the apparatus, which might indicate a deteriorated perception making them more vulnerable. The number of arm transitions was similar in MMI pups (4.9 transitions in MMI21 and 3.5 in MMI10 pups) and significantly lower than controls (12.3 transitions; *P* < 0.001; Figure 15D). These data most likely reflects increased anxiety-like in MMI21 pups, whereas in MMI10 pups other factors, caused by gestational hypothyroidism, such as deteriorated perception, should not be excluded.

## Discussion

This study shows that both developmental and early postnatal hypothyroidism in rats affect the density, percentage, ratio and size of VGluT1-ir and VGAT-ir boutons in the somatosensory cortex and hippocampal formation. Our data show that in the somatosensory cortex of MMI pups, VGluT1-ir bouton density is decreased in supra-granular layers, while VGAT-ir bouton densities are decreased compared to controls in all cortical layers except layer I. Regarding hippocampal formation, connectivity of the basic hippocampal trisynaptic loop is altered in MMI pups. In addition, both VGluT1-ir and VGAT-ir bouton density and size is decreased in the distal-inner molecular and granular layers of DG in MMI pups, reflecting a reduction in the total number of connections and transmitter release, with a consequent alteration of the total information flow in the somatosensory cortex and hippocampal formation. In MMM21 pups, both the acoustic startle response amplitude and prepulse inhibition percentage are reduced, while the percentage of time spent in open arms is increased. In contrast, MMI10 pups show less careful behavior in the elevated plus maze than MMI21 and C pups, which suggests that they might have profoundly affected hearing, memory and sensory perception. The behavior of MMI21 pups might also have been affected by alterations in hearing, memory and sensory perception, however their response to different sound intensities indicated a certain degree of hearing and they did not fall from open arms, as MMI10 pups frequently did. This suggests that MMI21 pups might show a certain degree of attention deficit and altered anxiety-like behavior.

### The experimental design

In rodents, the postnatal development of VGluT1 and VGAT expression has been studied using Western blots, immunocytochemistry and electron microscopy (Minelli et al., 2003a,b). These studies showed that the expression of VGluT1 and VGAT in the somatosensory cortex is weak at birth and progressively increases, reaching adult values at P20–30 (Minelli et al., 2003b) and at P15–20 (Minelli et al., 2003a) respectively, which matches the period of lactation of the pups. In rats, VGluT1 and VGAT immunolabeling has also been used to study the interaction between glutamatergic and GABAergic synapses (Merchán-Pérez et al., 2009). VGAT immunolabeling has proven to be a good marker for GABAergic boutons and has been used for the analysis of complex perisomatic formations on pyramidal neurons in several cortical areas in humans (Blázquez-Llorca et al., 2010). As such we have used deconvoluted confocal images for the quantification of VGluT1-ir and VGAT-ir bouton density and size in C and MMI pups at P50 because at this age the adult levels of VGluT1 and VGAT expression are reached (Minelli et al., 2003a,b). In addition, the principal excitatory neurons in the neocortex, DG and CA are glutamatergic, whereas all the inhibitory interneurons in the neocortex (DeFelipe et al., 2013) and the principal inhibitory interneurons in the hippocampal formation (basket and calretinin-ir) are all GABAergic (Freund, 2003). Basket (parvalbumin-ir and cholecystokinin-ir) interneurons synapse on principal neurons, whereas calretinin-ir interneurons selectively synapse on interneurons (Freund, 2003).

The postnatal maturation of the cerebral cortex is comparatively longer in humans than in rats (Marín-Padilla, 1978). MMI21 rats mimic the condition of congenital hypothyroidism, which causes functional alterations in the cerebral cortex and impairs cognitive development in humans (O'Callaghan et al., 1995; Kester et al., 2004; Rovet and Simic, 2008; Williams and Hume, 2008; Willoughby et al., 2014). However, similarities can be established considering that basic events of cortical maturation (such as axonal sprouting and pruning, myelination and synaptogenesis) and function are controlled by evolutionary preserved T3-regulated genes (see below; Morte et al., 2010; Berbel et al., 2014; Chatonnet et al., 2015). Our data show that early postnatal hypothyroidism alters VGluT1-ir and VGAT-ir bouton density in the hippocampus and somatosensory cortex, leading to an abnormal flow of information into these cortical areas (see next caption).

### Effects on connectivity

The cerebral cortex, and in particular the somatosensory cortex and hippocampus, is a laminated structure extremely sensitive to changes in the arrangement of neurons and the balance between excitatory to inhibitory inputs (Mountcastle, 1995; Klausberger and Somogyi, 2008; Rakic, 2009). A normal pattern of connections is required for a normal function of the cerebral cortex and it depends on many factors, among these the activation/inhibition of neuronal signaling pathways by chemo-attractive/repulsive signals and the functional activity of the axons (Skutella and Nitsch, 2001; Lewis et al., 2013; Sotelo and Dusart, 2014). Neuronal atrophy and abnormal connectivity were described in the hippocampus of developmentally hypothyroid rats several decades ago (Rami et al., 1986). Recent data have shown that the expression of genes involved directly or indirectly in the growth, path-finding and maturation of axons, as well as synaptic establishment and function are regulated by T3 at the transcriptional level. For instance, the Gsk3β-Crmp2 pathway is affected in the developmentally hypothyroid and hypothyroxinemic rat hippocampus (Wong and Leung, 2001; Wei et al., 2013). Perinatal hypothyroidism alters the expression of Gap-43, Sema3A, and Camk4-Creb pathways (Morte et al., 2010; Navarro et al., 2014) and the Erk1/2-Creb pathway is altered in the hippocampus of pups born to late hypothyroid dams (Lu et al., 2005; Berbel et al., 2010). BDNF expression is abnormal in the hippocampus of adult hypothyroid rats (Cortés et al., 2012), and it is involved in regulating the translational expression of VGluT1 in cultured hippocampal neurons (Chakraborty et al., 2012; Melo et al., 2013), in the regulation of FMRP synaptic function (Nishimura et al., 2007; Castrén and Castrén, 2014). In addition, the sonic hedgehog (Shh) signaling pathway, which is involved in the formation of microcircuits in the cerebral cortex (Harwell et al., 2012), is altered in embryonic and adult hypothyroid rats. In adults, the expression of the Shh-receptor Smo is downregulated in the DG (Desouza et al., 2011).

Ramón y Cajal (1901–1902) was the first to describe the basic trisynaptic excitatory loop between three areas of the hippocampal formation (entorhinal cortex, DG and CA). DG receives its main input from layer 2 of the entorhinal cortex via the perforant pathway (the lateral region projects to the distal-outer molecular layer whereas the medial region projects to the distal-inner molecular layer). DG granule cells project mostly to the proximal apical dendrites (stratum lucidum) of CA3 pyramidal cells, which in turn, project to ipsilateral apical dendrites (stratum radiatum) of CA1 pyramidal cells through the Schaffer collaterals. There is also a projection from layer 2 entorhinal cortex to the strata radiatum and lacunosum-moleculare of CA3 (to the medial and distal apical dendrites of pyramidal cells, respectively), and from layer 3 of the entorhinal cortex to the stratum lacunosum-moleculare of CA1 (to the distal apical dendrites of pyramidal neurons) (Amaral and Witter, 1995). In addition to the basic sequential trisynaptic loop described above, there is also a dense associative excitatory network interconnecting CA3 to ipsilateral CA3 and DG through recurrent connections (Amaral and Witter, 1995; Lisman et al., 2005), as well as to contralateral CA3 and CA1 through commissural connections (Amaral and Witter, 1995). Our findings show that the excitatory trisynaptic loop is altered in MMI pups. Decreased VGluT1-ir bouton density was found in (i) the distal-inner molecular layer of the DG (receiving afferents from layer 2 of the entorhinal cortex medial region; Figures 7B,C; asterisks), (ii) the stratum lucidum of CA3 (receiving afferents from DG; Figures 9B,C; arrowheads), and (iii) the strata lacunosum-moleculare (receiving afferents from layer 3 entorhinal cortex) and the proximal radiatum of CA1 (receiving afferents from CA3; Figures 11B,C). These alterations have several physiological implications. For instance, it has been reported that increased granule cell activity suppresses the overall excitability of the CA3 recurrent system (Acsády et al., 1998). Our results show a significant reduction of VGluT1 immunolabeling in the distal-inner molecular layer of the DG in MMI pups, and according to previous findings (Frotscher, 1989; Acsády et al., 1998), this might result in decreased DG granule cell activity and a decrease in GABAergic inhibition in CA3, producing increased CA3 recurrent excitability. Alterations in the basic excitatory trisynaptic loop might have an adverse effect on the encoding and recall of memory sequences (Squire, 1982; Lisman, 1999; Lisman et al., 2005; Bahar et al., 2011). In addition, we have found decreased MMI VGluT1-ir bouton density in all DG and CA layers receiving afferents from the entorhinal medial and lateral regions, which suggests abnormal cytoarchitecture of the entorhinal cortex and consequently aberrant connections with the hippocampal formation (Sloviter et al., 2012). Frotscher et al. (1997) showed that sprouting in the hippocampus after entorhinal cortex lesion is layer specific and that there is limited translaminar sprouting. Our data support these findings, since the decreased VGluT1-ir bouton density in the distal-inner molecular layer of the DG shows little sprouting from adjacent layers. The entorhinal cortex cytoarchitecture and connectivity in developmental and early postnatal hypothyroid rats should be explored in future studies, given the importance of the entorhinal cortex in both item and contextual discrimination (Hunsaker et al., 2013).

In the somatosensory cortex, a significant reduction of VGluT1-ir bouton density was seen in layers I, II-III and IV of MMI pups, while VGAT-ir bouton density decreased in all cortical layers but layer I. The decreased VGluT1-ir bouton density in layer IV of MMI10 pups is in agreement with previous results (Ausó et al., 2001). This study showed that the thalamic afferents to layer IV were smaller in developmentally hypothyroid pups. The number and length of terminal branches of thalamic axons, and the number of boutons were significantly less than in control pups (Ausó et al., 2001). These data might in part explain the reduction of VGluT1-ir bouton density found in layer II–III of MMI pups (asterisks in Figures 13F,K, 14A,C). However, reduced axonal development of neurons projecting to layer II–III (mostly layer IV neurons) should not be excluded. Interestingly, the VGluT1-ir bouton percentage decreased in layer II–III and increased in layer VI of MMI pups. These data might reflect an altered radial migration as described in the somatosensory (Berbel et al., 2001) and auditory (Lucio et al., 1997) cortices, and corpus callosum (Goodman and Gilbert, 2007) of thyroid hormone deficient pups.

### Altered excitatory-inhibitory balance. funtional implications

Several genes involved in the maturation of axons and dendrites, synaptogenesis and neurotransmission have been found to be regulated by T3 at the transcriptional level (Morte et al., 2010; Chatonnet et al., 2015) and mutated in autistic patients (Berbel et al., 2014). For example, genes found to be mutated in ASD include: (i) *CNTN4* that codes for contactin-4 (Zuko et al., 2013) and *BDNF* (Nishimura et al., 2007); (ii) *CALB1* and *PVALB* that code for calbindin-1 28 kDa and parvalbumin, respectively (Stoner et al., 2014); (iii) *GABRB3* that codes for GABA_A_β3 receptor (Fatemi et al., 2009, 2011; Stoner et al., 2014); and (iv)*HOMER1*, which codes for a postsynaptic density-localized scaffolding protein (Kelleher et al., 2012). Homer and Shank proteins interact to form an extended polymeric platform required for the recruitment and assembly of synaptic proteins and for the structural integrity of dendritic spines (Gilbert and Sui, 2006; Betancur and Buxbaum, 2013). This interaction has been shown to promote morphological and functional maturation of dendritic spines (Kelleher et al., 2012; Betancur and Buxbaum, 2013). Other T3-regulated genes involved in neurotransmition are *ANXA6* that codes for annexin 6 and is involved in Ca^++^ homeostasis (Sánchez-Ponce et al., 2011), and *ANK3* that codes for ankyrin 3 and is associated with the spectrin-actin neuronal skeleton binding to voltage gated Na^+^ channels (Bi et al., 2012). Both code proteins that are found in the initial segment of the axon. Moreover, mutations of the T3-regulated *KCNJ10* gene (Morte et al., 2010), that codes for astrocyte ATP-sensitive inward rectifier K^+^ channel 10, and is involved in the extracellular homeostasis of K^+^, have been also found in epileptic and ASD patients (Bockenhauer et al., 2009; Sicca et al., 2011).

These genetic alterations are subjacent to morphological alterations found in hypothyroid rats and might also explain the abnormal VGluT1-ir and VGAT-ir bouton density observed in MMI pups. Previous studies reported atrophied thalamic afferents to the barrel cortex in the developmental hypothyroid rat (Ausó et al., 2001), and reduced mossy fiber zinc density in developmental and postnatal hypothyroid rats (Savage et al., 1992; Madeira and Paula-Barbosa, 1993). In pups born to late hypothyroid dams, the Zn-positive area of the stratum lucidum was reduced by 41.5%. Zinc transporter-3 (ZnT3), Erk1/2 and Creb expression were also reduced (Berbel et al., 2010). Zinc and ZnT3 regulate memory formation, acting through the Erk1/2 signaling pathway (Mott and Dingledine, 2011). We have found a decreased VGluT1-ir bouton projection area in the stratum lucidum of CA3 in MMI pups compared with C pups. In addition, changes in VGAT-ir bouton density have been observed in: (i) MMI granular and proximal molecular layers, and in MMI10 distal-inner molecular layer of the DG (Figures 5B,C), (ii) MMI10 strata lucidum, radiatum and lacunosum-moleculare of CA3 (Figures 8B,C), and (iii) MMI stratum proximal radiatum of CA1 (Figures 11B,C). Perisomatic inhibitory boutons in the hippocampus innervate the somata, proximal dendrites, and axon initial segments of granule and pyramidal neurons (Freund, 2003; Klausberger and Somogyi, 2008), and control the pattern and timing of neuronal output, resulting in a synchronization of their response (Freund, 2003). The loss of these synchronized responses of hippocampal neurons does alter the hippocampus-dependent working memory (Kesner, 2013). Clinically, working memory impairment is important because it is strongly associated with poor academic achievement (Gathercole et al., 2006), which is common in hypothyroidism, hypothyroxinemia, ADHD and ASD. In particular, impaired working memory has been found recently in hypothyroid patients through the use of functional magnetic resource imaging (He et al., 2011). Interestingly, the T3-regulated gene *NR4A1* that codes for the transcription factor Nurr77 has an important role in the maintenance of long-term synaptic plasticity, consistent with the consolidation of long-term hippocampus-dependent memory (Bridi and Abel, 2013).

VGluT1-ir perisomatic boutons are abundant in neocortical pyramidal neurons, (Minelli et al., 2003b; Alonso-Nanclares et al., 2004) whilst being very scarce in the hippocampal formation. In the granular and pyramidal layers of C and MMI rats we have found a very low density of VGlutT1-ir boutons. In contrast, the density of VGAT-ir perisomatic boutons is high in both neocortex and hippocampus. Increased perisomatic inhibition results in increased control of the synchronized firing of hippocampal principal neurons (Freund, 2003; Klausberger and Somogyi, 2008; Isaacson and Scanziani, 2011). Thus the decreased VGAT-ir bouton density found granular layer in DG of MMI pups might result in decreased perisomatic inhibition, and a consequently asynchronized control of principal neuron firing. Asynchrony and hyperfunction of the cerebral cortex may be the pathophysiological foundation of ADHD and relate to the deficits of working memory processing and to impulsive symptoms (Li et al., 2014a). *In vitro* studies show that T3 regulates axonal length and spine density by spontaneous activity-dependent mTOR and trkB signaling, interacting with BDNF (Westerholz et al., 2013). Neurotrophins are well-known as mediators of activity-dependent effects, and might be responsible for the plastic changes in response to chronic activity deprivation found in the recurrent excitatory circuits of CA3 (Mitra et al., 2012). Thus, early T3 action contributing to the maturation of the excitatory-inhibitory balance in cortical circuits might be regulated by spontaneous activity-dependent mTOR and trkB signaling, interacting with the expression of neurotrophic factors. Furthermore, there is increasing evidence for a disturbed excitatory - inhibitory balance in ASD (Coghlan et al., 2012; Tebartz van Elst et al., 2014).

The activity of GABAergic interneurons plays a crucial role in the regulation of cerebral cortex function. We have found a decreased VGAT-ir bouton density in the hippocampus and somatosensory cortex of MMI pups. The decreased density of boutons might be due to decreased density of GABAergic neurons and the atrophy of their axonal arbors. The parvalbumin immunostaining pattern is severely altered in the neocortex (Berbel et al., 1996; Gilbert and Sui, 2006; Wallis et al., 2008) and hippocampus of hypothyroid rats (Guadaño-Ferraz et al., 2003; Venero et al., 2005; Gilbert and Sui, 2006; Sawano et al., 2013), showing atrophied basket and chandelier formations. Decreased cell density of parvalbumin-positive neurons in the somatosensory cortex and hippocampal formation of early postnatal hypothyroid rats has been reported (Gilbert et al., 2007) and the cortical tangential migration of GABAergic neurons from lateral to medial cortical areas was found to be abnormal in hypothyroxinemic rats (Cuevas et al., 2005). Interestingly, decreased GABAergic cell density has been found in the hippocampus of autistic humans at ages ranging from 13 to 63 years old (Lawrence et al., 2010). Using neuronal cell and organotypic cultures, it has been shown that T3 promotes GABAergic interneuron development, along with regulating the initial steps of the functional network synapse formation and appearance of early synchronized network activity (Westerholz et al., 2010). Electrophysiological recordings in CA1 show decreased long term potentiation (LTP) in developmental (Gilbert et al., 2007; Opazo et al., 2008; Wang et al., 2014) and postnatal hypothyroid rats (Alzoubi et al., 2009). Decreased Na^+^ currents in cultured hippocampal neurons (Hoffmann and Dietzel, 2004), and numbers of bursting CA1 cells and spikes per burst, resulting from altered low-threshold Ca^2+^ currents (Sánchez-Alonso et al., 2010) have been found in developing hypothyroid rats. In addition to the reduced excitability of hippocampal neurons observed in these studies, the reduced glutamatergic input to supragranular layers of DG suggested by the smaller VGluT1-ir bouton density and size observed, and the decrease in inhibitory VGAT-ir boutons in the granular layer suggests an altered information flow throughout the hippocampal pathways as a result of hypothyroidism.

The etiopathology of seizures is little known although it has been associated with excessive or abnormal synchronous neuronal activity in the neocortex and hippocampus (Marco et al., 1997; DeFelipe, 1999; Alonso-Nanclares et al., 2011). These authors have found reduced perisomatic inhibition of principal neocortical neurons. Our data show a reduction of VGAT-ir bouton density and size in somatosensory layer VI of MMI pups, and previous studies have reported a decrease of parvalbumin immunoreactivity in the auditory cortex of adult hypothyroid rats (Berbel et al., 1996). Interestingly, seizure-susceptibility was studied in adult rats treated with goitrogens, such as propylthiouracil and MMI during pregnancy and lactation (Van Middlesworth and Norris, 1980; Ausó et al., 2004; Pacheco-Rosado et al., 2005; Giné et al., 2010) and in thyroid hormone receptor mutant mice (Ng et al., 2001; Hadjab-Lallemend et al., 2010). Electrocochleograms performed on 225 adult rats (treated with propylthiouracil from P0 to P19) showed that 89% of these were sensitive to audiogenic seizures with a loss of 60 dB hearing sensitivity (Van Middlesworth and Norris, 1980). It has been reported that an aberrant connectivity between the entorhinal cortex and the dentate gyrus causes epileptiform discharges in granule cells and might cause clinical seizures (Pickett and London, 2005; Sloviter et al., 2012). In agreement, our results show that excitation and inhibition become unbalanced in the somatosensory cortex and hippocampal formation. In particular, the connectivity between the entorhinal cortex and the dentate gyrus is abnormal in MMI pups.

### Altered behavior

The association of hypothyroid rat behavior with cerebral cortex alterations is mainly based on tests oriented toward (i) locomotor functional excitability and seizure susceptibility (Van Middlesworth and Norris, 1980; Ng et al., 2001; Ausó et al., 2004; Wallis et al., 2008), and (ii) learning, attention and memory deficits (Negishi et al., 2005; Venero et al., 2005; Gilbert et al., 2007; Opazo et al., 2008; Berbel et al., 2010; Gilbert and Lasley, 2013). In open field test, *TR*α1^−/−^ mice showed lower rearing and increased freezing levels than wild-type mice (Guadaño-Ferraz et al., 2003). Transient maternal and fetal hypothyroxinemia in pregnant rats at the beginning of fetal corticogenesis affects the protein composition of the postsynaptic density of synapses in CA1, and the spatial learning of the offspring (Opazo et al., 2008). In studies using adult hyperthyroid rats, it was found that LT4 treatments have no influence on 2-way avoidance, despite increased mossy fiber density in the CA3 stratum oriens (Lipp et al., 1984, 1988), and no differences between hypothyroid and control rats in the passive light avoidance test have been seen (Tamasy et al., 1986).

Our findings show that the excitatory trisynaptic loop is altered in MMI pups. In particular the decreased VGluT1-ir bouton density found in the stratum lucidum of CA3 and the proximal radiatum of CA1 reflects an abnormal connection between CA3 and CA1 that most likely will affect associative learning in MMI pups. In mouse behavioral studies, the response of the CA3 to CA1 synapses seems to be modulated during associative learning, and both processes (synaptic response and associative learning) are prevented by experimental LTP or N-methyl-D-aspartate (NMDA)-receptor inactivation (Gruart et al., 2006). Both associative learning and memory have been shown to be regulated by thyroid hormones. In mice treated with ethylcholine mustard aziridinium ion, that causes a selective reduction of choline acetyltransferase activity and glutamate level in the hippocampus, it has been found that LT3 administration (once daily for 6 days) reduces the deficiency in working memory performance and reverses the decreased acetylcholine and glutamate levels in the hippocampus (Abe et al., 1992). Postnatal LT4-induced hyperthyroidism also improved spatial learning and working memory in mice at P90 (Crusio and Schwegler, 1991; Schwegler et al., 1991). In TRα knockout mice impaired spatial learning and memory has been found (Wilcoxon et al., 2007). In addition, developmental, perinatal and adult hypothyroidism induced a decreased LTP in CA1 (Niemi et al., 1996; Sui and Gilbert, 2003; Gilbert, 2004), whereas long term depression (LTD) was not affected (Sui and Gilbert, 2003; Gilbert, 2004). It has been reported that LT3 and LT4 treatment starting at P80, of rats thyroidectomyzed at P61, does not lead to a recovery of LTP (Fernández-Lamo et al., 2009). Decreased LTP and increased LTD (Vara et al., 2003), associated with increased hyperactivity (Akaike et al., 1991; Vara et al., 2003) was found in early and late postnatal hypothyroid rats.

In mammals, prepulse inhibition of the acoustic startle response studies the reduction of the response by a weak pulse preceding the startle pulse, and is used to measure sensorimotor gating mechanisms (Graham, 1975; Norris and Blumenthal, 1996; Koch, 1999). Deficient sensorimotor gating, as reflected by disrupted prepulse inhibition, exists in several neuropsychiatric disorders such as schizophrenia (Braff et al., 2001; Hamm et al., 2001). The acoustic startle response of mammals is mediated by a relatively simple neuronal circuit located in the lower brainstem (Koch, 1999), however some studies have established a relation between prepulse inhibition and hippocampus (Zhang et al., 2002; Daenen et al., 2003; Howland et al., 2004). For instance, it has been reported that the temporary inactivation by tetrodotoxin or inhibition by muscimol of the dorsal or ventral hippocampus can impair prepulse inhibition. Muscimol inhibits local neuronal activity by acting on the GABA_A_ receptor while TTX is inactivating the signal conduction of fibers of passage by blocking sodium channels (Zhang et al., 2002). The startle amplitude was reduced 45.6% in MMI21 and 79.8% in MMI10 pups compared to C pups, which would suggest severely impaired hearing in MMI10 pups. The reduced percentage of prepulse inhibition found in MMI21 pups, which is stimulus intensity dependent could likewise, might result from a decreased neuronal activity due to decreased voltage-dependent ion currents in concert with decreased neurotransmitter release in the hippocampus and somatosensory cortex, and reflects a degree of attention deficit.

Hearing loss has been reported in hypothyroid rats and the degree of hearing loss depends on the timing and severity of thyroid hormone deficiency. In humans, hearing loss has been found in cretinism (Trotter, 1960; DeLong et al., 1985; Morreale de Escobar et al., 2000), congenital in congenital hypothyroidism (Vanderschueren-Lodeweyckx et al., 1983; Rovet et al., 1996), and in Refetoff syndrome (Refetoff et al., 1967). Deafness may be complete in as many as 50% of patients suffering cretinism, in which no cochlear or brain stem response is seen (Halpern et al., 1989; Ma et al., 1989). Cochlear impairment and hearing loss with a decrease in hearing threshold by 20–60 dB was found in five different types of thyroid hormone receptor mutants (Ng et al., 2001; Rüsch et al., 2001). Knipper et al. (2000) showed that thyroid hormone deficiency during the first two postnatal weeks causes irreversible damage in the Corti's organ which increases the hearing threshold of rat pups by up to 60%. The hearing loss was later found to be associated with prolonged spiking activity of inner hair cells of the organ of Corti, due to elevated Ca^2+^ currents and the absence of voltage-activated K^+^ currents (Brandt et al., 2007). We have used 74, 82, and 90 dB stimulus intensities that are within the normal range of hearing frequencies in postnatal hypothyroid pups (Van Middlesworth and Norris, 1980), and found that the startle amplitude and percentage of prepulse inhibition was reduced in MMI pups compared to control. MMI10 pups may be unable to respond to sound stimuli, but in contrast, MMI21 pups show hearing responses.

The elevated plus-maze has been used frequently as a tool to assess the anxiogenic effects of drugs (Lister, 1990), and there is evidence that the hippocampus may be involved in anxiety-related functions (Engin and Treit, 2007). When infused into the dorsal hippocampus, the directly acting GABA_A_ agonist muscimol produces anxiolytic effects in the plus-maze device, whereas bicuculline, a selective GABA_A_ receptor antagonist, does not (Rezayat et al., 2005). In summary, the data thus far suggest that the dorsal hippocampus plays some role in anxiety, independently of its learning and memory-related functions (Engin and Treit, 2007). The behavior displayed in the elevated plus-maze test represents a combination of exploratory and avoidance behaviors, as well as general activity, all of which are influenced by both genetic and environmental factors (Carobrez and Bertoglio, 2005). The percentage of time spent in open arms increased in MMI pups compared to controls. However, the interpretation of elevated plus-maze results in hypothyroid rats is puzzling. As mentioned above, hypothyroidism causes severe behavioral alterations, among these impaired spatial learning and memory loss (Wilcoxon et al., 2007). Thus, hypothyroid rats might have loss in orientation and the loss of memory might lead to “forgetting” that a situation may be dangerous. This might explain the risk-taking behavior of MMI10 pups, so they usually walked until the end of the open arm and fell down. In MMI21 rats, impaired spatial learning and memory loss might also altered their behavior in the elevated open arms. However, they showed a non-risky behavior non-risky behavior, reflecting an high degree of anxiety.

## Conclusions

In summary, we have found that the distribution, ratio and size of VGluT1-ir and VGAT-ir boutons in the somatosensory cortex and hippocampal formation are abnormal in pups hypothyroid from E11 or E21 to P50. In MMI pups the VGAT-ir/VGluT1-ir ratio is increased in DG and decreased in CA3, CA1, and somatosensory cortex. In addition, immunoreactive boutons were smaller in size, resulting in an overall reduction of synaptic transmission in all areas. These alterations affect both the intrinsic and extrinsic flow of information in the hippocampus and neocortex. MMI pups showed alterations in behavior, with prepulse inhibition percentage and acoustic startle response amplitude being reduced and time spent in elevated plus-maze open arms increased. Further studies of excitatory and inhibitory balance will increase our understanding of memory loss and neurocognitive alterations that might occur in developmental and early postnatal hypothyroidism. Despite the differences between rodents and humans, our data may help to better understand the comorbidity of thyroid associated diseases and human psychiatric disorders such as ADHD and ASD, which might share similar alterations during development and maturation of neural circuits.

## Author contributions

The conception, design and draft of the work were carried out by PB. DN, MA, MG and PB contributed to the acquisition, analysis and interpretation of histological data; MJO contributed to thyroid hormone determinations; FN and JM contributed to the acquisition, analysis and interpretation of behavior tests. All authors contributed discussing the results and writing specific parts of the manuscript and give the final approval of the manuscript.

### Conflict of interest statement

The authors declare that the research was conducted in the absence of any commercial or financial relationships that could be construed as a potential conflict of interest.
