# Supplementary material for: Gestational and early postnatal hypothyroidism alters VGluT1 and VGAT bouton distribution in the neocortex and hippocampus, and behavior in rats
Source: Front Neuroanat. 2015 Feb 17;9:9. doi: 10.3389/fnana.2015.00009 (PMC4330898; doi:10.3389/fnana.2015.00009)
Supplement: Supplementary file 1 [file Table1.PDF]

Table 1. VGluT1-ir and VGAT-ir bouton density, percentage and area in DG.

	VGluT1			VGAT		
	**Control**	**MM21**	**MMI10**	**Control**	**MM21**	**MMI10**
**Bouton no./$10^4 \mu m^2$**						
Molecular (d-o)	1716 ± 90	1690 ± 65	1563 ± 112	854 ± 73	831 ± 85	892 ± 44
Molecular (d-i)	1693 ± 50	1446 ± 58	1245 ± 56	781 ± 63	822 ± 41	874 ± 80
Molecular (p)	1574 ± 63	1577 ± 83	1511 ± 72	722 ± 42	713 ± 37	877 ± 93
Granular	112 ± 26	117 ± 16	105 ± 14	907 ± 81	846 ± 80	712.7 ± 54
Hilus	1280 ± 120	1164 ± 37	1062 ± 53	553 ± 80	502 ± 65	475 ± 38
**Bouton %**						
Molecular (d-o)	28.1 ± 1.4	30.0 ± 0.9	31.8 ± 1.8	21.9 ± 1.8	21.9 ± 1.7	24.3 ± 1.1
Molecular (d-i)	37.3 ± 0.8	31.4 ± 1.1	28.3 ± 1.4	26.9 ± 1.9	26.8 ± 1.9	26.6 ± 1.5
Molecular (p)	21.5 ± 0.8	25.1 ± 1.0	26.6 ± 0.7	15.5 ± 1.1	17.0 ± 06	20.7 ± 2.3
Granular	2.2 ± 0.5	2.5 ± 0.3	2.4 ± 0.3	28.3 ± 2.3	27.2 ± 2.1	21.8 ± 1.4
Hilus	10.9 ± 0.9	11.0 ± 0.4	10.9 ± 0.4	7.4 ± 0.9	7.1 ± 0.9	6.6 ± 0.4
**Bouton % in each layer**						
Molecular (d-o)				33.2 ± 2.4	32.9 ± 1.9	36.4 ± 2.1
Molecular (d-i)				31.5 ± 1.4	36.3 ± 1.4	41.2 ± 2.3
Molecular (p)				31.5 ± 1.5	31.1 ± 1.3	36.7 ± 3.0
Granular				88.9 ± 2.7	87.8 ± 1.7	87.1 ± 1.5
Hilus				30.2 ± 3.9	30.0 ± 3.1	30.9 ± 2.0
**Bouton area (μm^2)**						
Molecular (d-o)	0.21 ± 0.03	0.20 ± 0.03	0.17 ± 0.02	0.19 ± 0.03	0.19 ± 0.02	0.18 ± 0.01
Molecular (d-i)	0.17 ± 0.02	0.17 ± 0.02	0.15 ± 0.01	0.19 ± 0.01	0.19 ± 0.01	0.18 ± 0.01
Molecular (p)	0.23 ± 0.04	0.24 ± 0.02	0.21 ± 0.02	0.20 ± 0.04	0.20 ± 0.01	0.23 ± 0.01
Granular	0.28 ± 0.03	0.26 ± 0.05	0.20 ± 0.02	0.33 ± 0.06	0.32 ± 0.02	0.29 ± 0.01
Hilus	0.34 ± 0.03	0.33 ± 0.02	0.27 ± 0.03	0.26 ± 0.06	0.26 ± 0.03	0.23 ± 0.01

p: proximal; d-o: distal-outer; d-i: distal-inner.
