# Supplementary material for: Gestational and early postnatal hypothyroidism alters VGluT1 and VGAT bouton distribution in the neocortex and hippocampus, and behavior in rats
Source: Front Neuroanat. 2015 Feb 17;9:9. doi: 10.3389/fnana.2015.00009 (PMC4330898; doi:10.3389/fnana.2015.00009)
Supplement: Supplementary file 2 [file Table2.PDF]

Table 2. VGluT1-ir and VGAT-ir bouton density, percentage and area in CA3.

	VGluT1			VGAT		
	**Control**	**MM21**	**MMI10**	**Control**	**MM21**	**MMI10**
**Bouton no./$10^4 \mu m^2$**						
Oriens	1588 ± 55	1545 ± 56	1532 ± 87	663 ± 79	640 ± 46	608 ± 54
Pyramidale	281 ± 48	272 ± 81	280 ± 53	787 ± 125	756 ± 72	707 ± 42
Lucidum	1025 ± 155	859 ± 126	724 ± 138	482±74	430±52	388±41
Radiatum	1588 ± 126	1514 ± 59	1389 ± 87	641 ± 60	626 ± 64	572 ± 43
Lacunosum-Moleculare	1425 ± 161	1394 ± 75	1225 ± 68	679 ± 65	640 ± 73	595 ± 41
**Bouton %**						
Oriens	34.1 ± 1.8	34.7 ± 1.0	35.3 ± 1.9	26.0 ± 1.9	26.2 ± 1.1	26.1 ± 1.6
Pyramidale	4.8 ± 0.8	4.9 ± 1.4	5.2 ± 1.0	24.3 ± 2.2	24.7 ± 2.0	24.4 ± 0.9
Lucidum	24.3 ± 2.9	17.3 ± 2.0	12.4 ± 2.0	20.9 ± 1.7	15.7 ± 2.0	12.1 ± 1.1
Radiatum	25.8 ± 1.3	31.6 ± 1.5	38.0 ± 1.8	19.1 ± 1.3	23.8 ± 1.9	29.1 ± 1.6
Lacunosum-Moleculare	11.0 ± 1.3	11.5 ± 0.6	9.1 ± 0.5	9.7 ± 1.3	9.6 ± 1.0	8.3 ± 0.7
**Bouton % in each stratum**						
Oriens				29.4 ± 2.8	29.2 ± 1.3	28.4 ± 2.2
Pyramidale				73.5 ± 3.9	73.9 ±5.9	71.8 ± 3.5
Lucidum				32.2 ± 4.9	33.5 ± 3.7	35.3 ± 5.0
Radiatum				28.8 ± 2.6	29.1 ± 1.7	29.2 ± 1.9
Lacunosum-Moleculare				32.4 ± 3.1	31.4 ± 2.7	32.7 ± 2.3
**Bouton area (μm^2)**						
Oriens	0.20 ± 0.02	0.19 ± 0.04	0.17 ± 0.02	0.28 ± 0.04	0.27 ± 0.05	0.28 ± 0.01
Pyramidale	0.62 ± 0.11	0.61 ± 0.08	0.47 ± 0.05	0.38 ± 0.07	0.37 ± 0.02	0.32 ± 0.05
Lucidum	0.89 ± 0.12	0.86 ± 0.13	0.70 ± 0.11	0.33 ± 0.07	0.32 ± 0.06	0.28 ± 0.01
Radiatum	0.21 ± 0.02	0.21 ± 0.04	0.18 ± 0.01	0.24 ± 0.03	0.23 ± 0.01	0.22 ± 0.01
Lacunosum-Moleculare	0.19 ± 0.02	0.20 ± 0.04	0.17 ± 0.01	0.23 ± 0.03	0.19 ± 0.02	0.20 ± 0.01
