# Supplementary material for: Gestational and early postnatal hypothyroidism alters VGluT1 and VGAT bouton distribution in the neocortex and hippocampus, and behavior in rats
Source: Front Neuroanat. 2015 Feb 17;9:9. doi: 10.3389/fnana.2015.00009 (PMC4330898; doi:10.3389/fnana.2015.00009)
Supplement: Supplementary file 3 [file Table3.PDF]

Table 3. VGluT1-ir and VGAT-ir bouton density, percentage and area in CA1.

	VGluT1			VGAT		
	Control	MM21	MMI10	Control	MM21	MMI10
**Bouton no./10^4 μm^2**						
Oriens	1528 ± 92	1507 ± 60	1511 ± 66	639 ± 32	636 ± 37	588 ± 55
Pyramidale	192 ± 23	208 ± 21	221 ± 21	795 ± 84	762 ± 35	757 ± 74
Radiatum (proximal)	1469 ± 55	1447 ± 82	1511 ± 43	600 ± 42	551 ± 23	520 ± 34
Radiatum (distal)	1267 ± 95	1325 ± 57	1489 ± 16	550 ± 78	553 ± 41	501 ± 7
Lacunosum-Moleculare	592 ± 54	710 ± 35	732 ± 50	632 ± 47	670 ± 43	691 ± 79
**Bouton %**						
Oriens	32.5 ± 1.5	32.7 ± 1.2	28.6 ± 0.9	25.7 ± 1.5	26.6 ± 1.1	22.6 ± 1.8
Pyramidale	1.4 ± 0.2	1.4 ± 0.1	1.6 ± 0.1	10.4 ± 1.0	10.8 ± 0.5	11.0 ± 1.3
Radiatum (proximal)	39.4 ± 1.4	37.0 ± 1.7	37.6 ± 0.9	30.4 ± 1.7	27.2 ± 1.0	26.5 ± 1.6
Radiatum (distal)	17.0 ± 1.2	16.9 ± 0.6	18.3 ± 0.3	13.9 ± 1.7	13.6 ± 1.2	12.7 ± 0.2
Lacunosum-Moleculare	9.7 ± 0.9	12.0 ± 0.4	13.9 ± 0.8	19.6 ± 1.7	21.8 ± 1.1	27.2 ± 2.1
**Bouton % in each stratum**						
Oriens				29.5 ± 1.2	29.7 ± 0.9	28.0 ± 2.6
Pyramidale				80.4 ± 2.8	78.6 ± 1.2	77.3 ± 1.8
Radiatum (proximal)				29.0 ± 1.9	27.6 ± 0.8	25.6 ± 1.1
Rad (distal)				30.2 ± 3.7	29.4 ± 1.5	25.2 ± 0.4
Lacunosum-Moleculare				51.7 ± 3.4	48.5 ± 1.4	49.0 ± 2.4
**Bouton area (μm^2)**						
Oriens	0.17 ± 0.02	0.18 ± 0.02	0.18 ± 0.02	0.23 ± 0.03	0.23 ± 0.01	0.25 ± 0.02
Pyramidale	0.24 ± 0.02	0.24 ± 0.02	0.20 ± 0.01	0.35 ± 0.04	0.34 ± 0.01	0.32 ± 0.01
Radiatum (proximal)	0.15 ± 0.01	0.16 ± 0.02	0.15 ± 0.01	0.20 ± 0.02	0.19 ± 0.02	0.18 ± 0.02
Radiatum (distal)	0.16 ± 0.01	0.17 ± 0.02	0.15 ± 0.01	0.20 ± 0.03	0.19 ± 0.02	0.18 ± 0.01
Lacunosum-Moleculare	0.17 ± 0.02	0.18 ± 0.01	0.16 ± 0.02	0.17 ± 0.02	0.18 ± 0.01	0.19 ± 0.01
