# Supplementary material for: Gestational and early postnatal hypothyroidism alters VGluT1 and VGAT bouton distribution in the neocortex and hippocampus, and behavior in rats
Source: Front Neuroanat. 2015 Feb 17;9:9. doi: 10.3389/fnana.2015.00009 (PMC4330898; doi:10.3389/fnana.2015.00009)
Supplement: Supplementary file 4 [file Table4.PDF]

Table 4. Density, percentage and area of VGluT1-ir and VGAT-ir boutons in CC.

	VGlut1			VGAT		
	**C**	**MM21**	**MMI10**	**C**	**MM21**	**MMI10**
**Bouton no./$10^4 \mu m^2$**						
I	1673 ± 71	1594 ± 68	1491 ± 61	517 ± 29	570 ± 51	503 ± 60
II-III	1655 ± 113	1530 ± 46	1338 ± 70	848 ± 57	810 ± 51	678 ± 52
IV	1494 ± 106	1453 ± 82	1219± 70	791± 68	760 ± 47	604 ± 37
V	1444 ± 116	1433 ± 60	1374 ± 87	655 ± 63	627 ± 31	562 ± 49
VI	1270 ± 96	12681 ± 58	1257 ± 51	843 ± 114	769 ± 50	655 ± 74
wm	113 ± 5	124 ± 6	172 ± 14	57 ± 1	62 ± 7	85 ± 6
**Bouton %**						
I	10.1 ± 1.0	9.5 ± 0.6	9.0 ± 1.0	6.0 ± 0.7	6.6 ± 1	6.5 ± 1.2
II-III	26.8 ± 1.6	24.8 ± 0.6	24.3 ± 2.4	26.4 ± 1.8	25.6 ± 1.2	26.4 ± 3.9
IV	8.1 ± 0.5	8.2 ± 0.3	7.3 ± 0.5	8.2 ± 0.7	8.4 ± 0.9	7.7 ± 0.7
V	25.3 ± 1.3	25.8 ± 1.5	26.8 ± 1.4	22.0 ± 2.2	22.0 ± 1.2	23.4 ± 1.1
VI	29.1 ± 1.8	31.0 ± 1.4	31.5 ± 1.7	36.8 ± 3.8	36.7 ± 2.0	34.9 ± 3.5
wm	0.6 ± 0.0	0.7 ± 0.0	1.1 ± 0.1	0.6 ± 0.0	0.7 ± 0.1	1.1 ± 0.2
**Bouton % in each layer**						
I				23.6 ± 1.3	26.3 ± 1.6	25.2 ± 1.8
II-III				33.9 ± 1.7	34.6 ± 1.3	33.6 ± 2.8
IV				34.6 ± 2.0	34.3 ± 2.1	33.2 ± 2.4
V				31.2 ± 1.8	30.4 ± 1.7	29.1 ± 2.4
VI				39.8 ± 3.8	37.8 ± 1.7	34.2 ± 1.9
wm				33.5 ± 1.5	33.2 ± 2.4	33.1± 1.5
**Bouton area (μm^2)**						
I	0.20 ± 0.02	0.20 ± 0.03	0.18 ± 0.02	0.20 ± 0.03	0.19 ± 0.02	0.2 ± 0.02
II-III	0.22 ± 0.02	0.21 ± 0.02	0.20 ± 0.03	0.28 ± 0.06	0.27 ± 0.03	0.24 ± 0.01
IV	0.22 ± 0.02	0.22 ± 0.02	0.19 ± 0.02	0.32 ± 0.05	0.33 ± 0.02	0.30 ± 0.03
V	0.25 ± 0.02	0.25 ± 0.02	0.21 ± 0.02	0.41 ± 0.06	0.38 ± 0.03	0.33 ± 0.05
VI	0.24 ± 0.02	0.23 ± 0.01	0.22 ± 0.03	0.31 ± 0.06	0.30 ± 0.03	0.28 ± 0.01
wm	0.23 ± 0.01	0.23 ± 0.02	0.23 ± 0.02	0.23 ± 0.01	0.23 ± 0.02	0.20 ± 0.03
